# Supplementary material for: Presence of complete murine viral genome sequences in patient-derived xenografts
Source: Nat Commun. 2021 Apr 1;12:2031. doi: 10.1038/s41467-021-22200-5 (PMC8017013; doi:10.1038/s41467-021-22200-5)
Supplement: Supplementary file 1 — Supplementary Information [file 41467_2021_22200_MOESM1_ESM.pdf]

Presence of Complete Murine Viral Genome Sequences in Patient-Derived Xenografts

Zihao Yuan, Xuejun Fan, Jay-Jiguang Zhu, Tong-Ming Fu, Jiaqian Wu, Hua Xu, Ningyan Zhang, Zhiqiang An, W. Jim Zheng

Supplementary Information

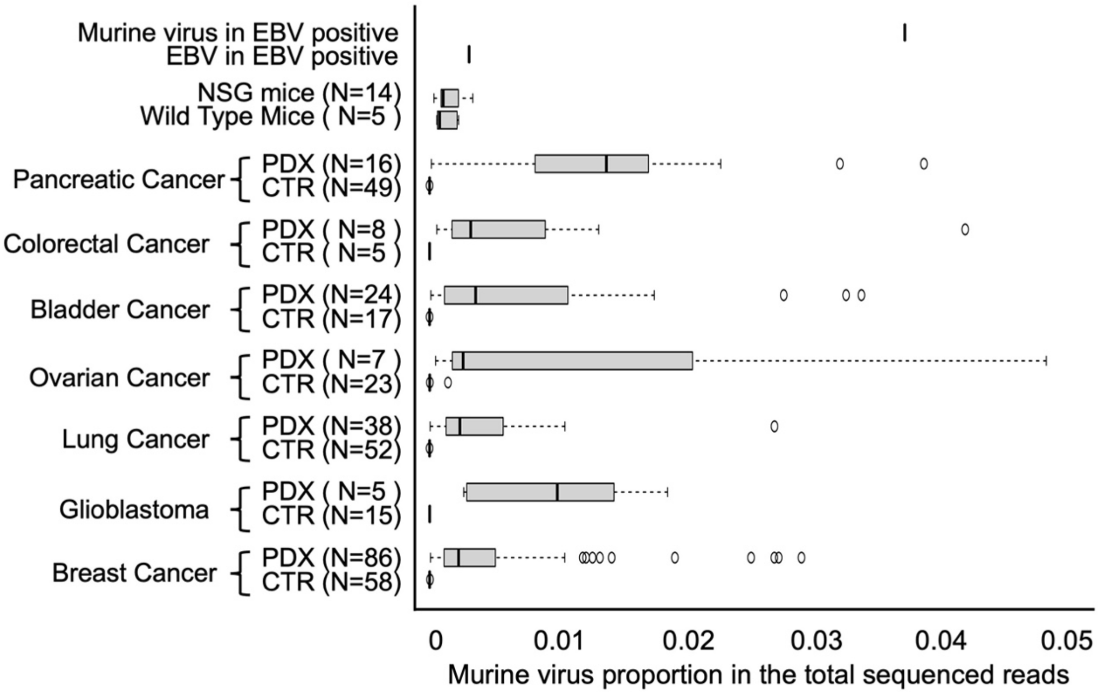

Supplementary Fig. 1 Percentages of murine virus reads in total sequenced reads from various PDX tumors, Box limits: 25th and 75th percentiles; center line: median; whiskers: 1.5x interquantile range from box limits; dots: outliers. Source data are provided in Source Data file.

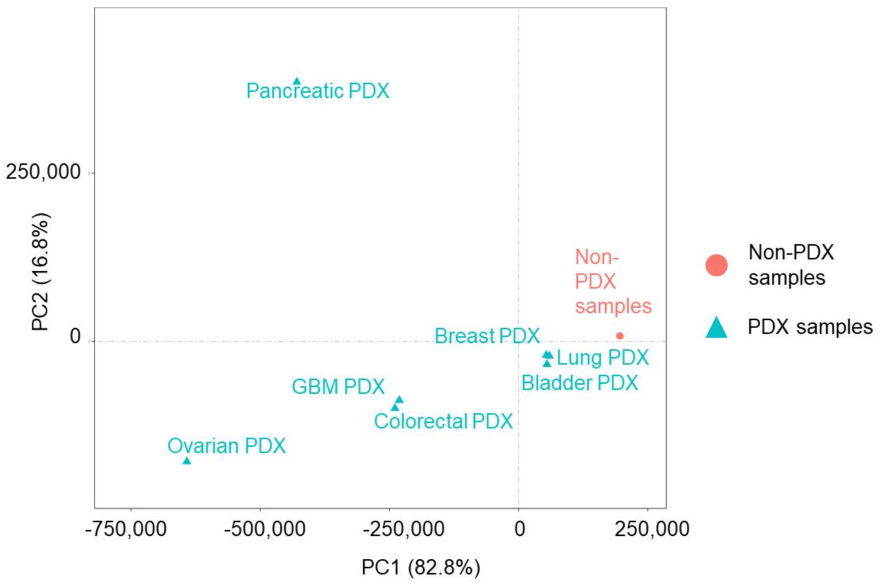

Supplementary Fig. 2 Principal component analysis (PCA) of the 7 PDX samples (Cyan) and 7 corresponding primary tumor samples (Red) based on the average number of reads of murine virus. The PCA analysis was conducted based on the average numbers of reads of murine virus in PDX and non-PDX groups in Omicshare (http://www.omicshare.com/tools/Home/Soft/pca).

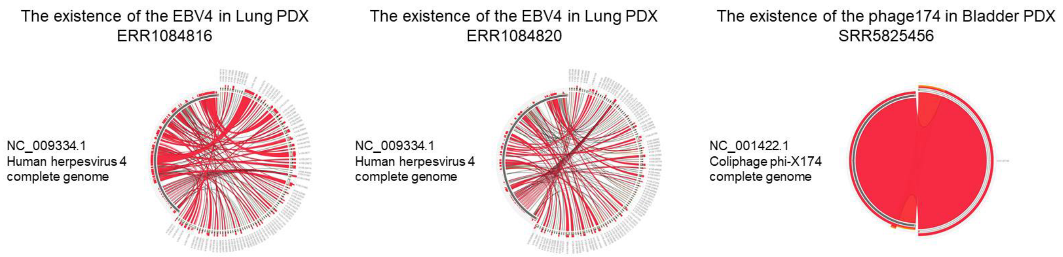

Supplementary Fig. 3. The assembly of the other representative viruses. The assembly of EBV in Lung PDX sample ERR1084816 (Left), ERR1084820 (Middle), and the phage X174 (Right) from Bladder PDX sample SRR5825456.

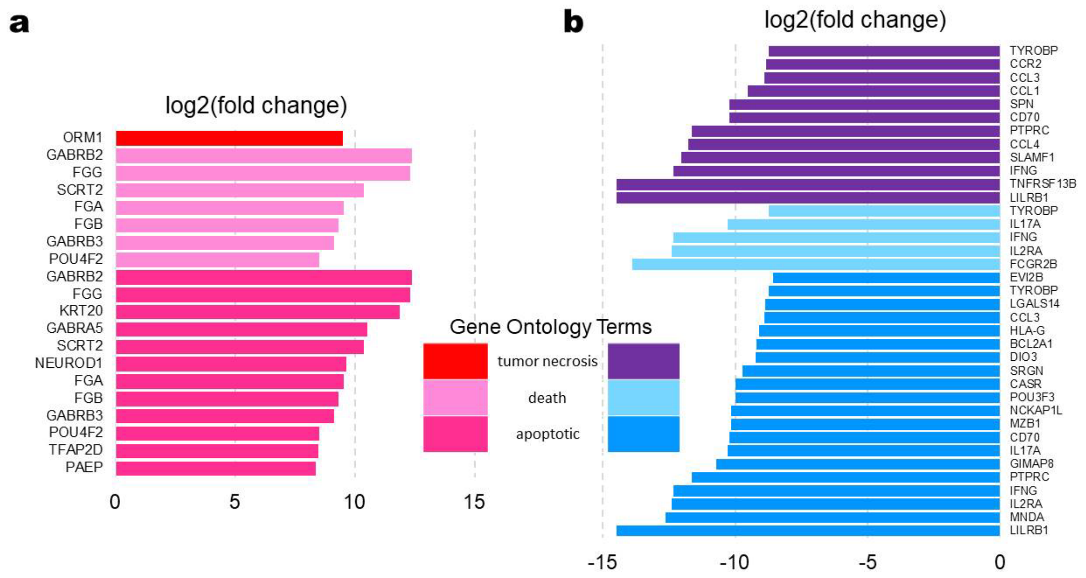

Supplementary Fig. 4. Gene Ontology Terms enrichment of the impact of murine viral infection on gene expression of PDX. Transcription profiles of lung cancer PDX samples with highest and lowest viral amounts were compared. Gene Ontology Terms were enriched among: **a** the top 200 up regulated genes related to tumor necrosis (Red), death (Pink), apoptotic (Magenta) and **b** the top 200 down regulated genes related to tumor necrosis (Purple), death (Light blue), apoptotic (Blue) in the top 5 PDX samples with the most murine viral infection. Source data are provided in Source Data file.

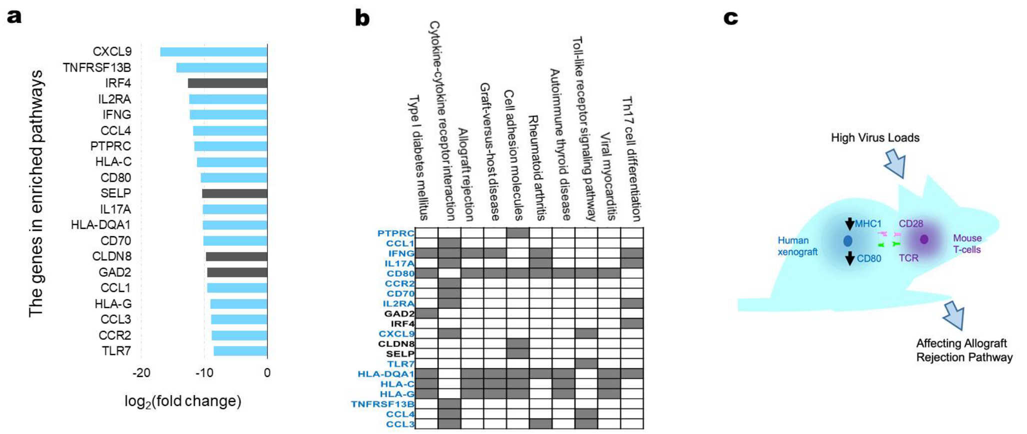

Supplementary Fig. 5 Impact of viral load on immune gene expression in PDX tumors. **a** Expression of down regulated genes from enriched pathways; immune related genes shown in blue. **b** Down regulated genes and the corresponding enriched pathways, with immune-related genes shown in blue. Filled boxes indicate the presence of the genes in the pathways. **c** High murine virus load can affect the allograft rejection pathway by down regulating the *MHC1* and *CD80* genes whose protein products are on the surface of human tumor tissues. Transcription profiles of bladder cancer PDX samples treated with pictilisib with highest and lowest viral amounts. Source data are provided in Source Data file.

Supplementary Table 1. 210 NIH-funded ongoing projects relevant to PDX. These projects were identified using the NIH Research Portfolio Online Reporting Tools (RePORT). The exact phrase "Patient derived Xenografts" were used as the search term to identify all the ongoing projects with this key phrase on March 25th, 2019.

T	Act	Project	FY	Admin IC	Funding IC	FY Total Cost by IC
1	I01	BX002746	2019	VA		
1	R01	CA224275	2018	NCI	NCI	$567,557
5	R01	NS088648	2018	NINDS	NINDS	$349,149
5	U01	CA213338	2018	NCI	NCI	$605,070
5	R01	CA196664	2018	NCI	NCI	$537,912
1	U54	CA233223	2018	NCI		$1,939,255
5	I01	BX003458	2019	VA		
5	R01	CA189209	2019	NCI	NCI	$352,155
5	R01	CA217180	2019	NCI	NCI	$385,203
1	F30	CA236302	2019	NCI	NCI	$46,765
1	R41	CA235796	2018	NCI	NCI	$299,552
1	U54	CA231652	2018	NCI		$1,713,215
5	R01	CA201069	2018	NCI	NCI	$410,606
1	I01	BX003840	2018	VA		
5	U24	CA204781	2018	NCI	NCI	$413,604
3	U01	CA214114	2018	NCI	NIA	$440,000
5	U01	CA214114	2018	NCI	NCI	$1,441,604
5	R01	CA195754	2017	NCI	NCI	$569,391
5	R00	CA201228	2018	NCI	NCI	$175,615
5	R01	CA148671	2018	NCI	NCI	$356,250
1	R43	CA228938	2018	NCI	NCI	$160,000
3	R01	CA186043	2019	NCI	NCI	$95,085
5	R01	CA186043	2019	NCI	NCI	$368,133
1	R01	CA217251	2018	NCI	NCI	$349,988
5	R01	CA221957	2019	NCI	NCI	$520,798
5	I01	BX002301	2019	VA		
5	R01	CA116021	2019	NCI	NCI	$361,688
1	R01	CA234720	2019	NCI	NCI	$445,546
5	P01	CA096832	2018	NCI		$138,791
5	U01	CA199215	2018	NCI	NCI	$570,759
5	R01	CA188575	2018	NCI	NCI	$349,457
1	R01	CA226746	2018	NCI	NCI	$493,327
1	F32	CA228316	2018	NCI	NCI	$61,174
5	K08	CA194268	2018	NCI	NCI	$169,020
1	K08	DE027730	2018	NIDCR	NIDCR	$168,156
5	R24	NS092940	2018	NINDS	NINDS	$381,112
5	R01	CA140985	2019	NCI	NCI	$318,829
5	P50	CA097190	2018	NCI		$153,941
1	R01	CA216101	2018	NCI	NCI	$670,933
1	ZIA	BC011736	2018	NCI	NCI	$370,592
1	ZIA	BC011848	2018	NCI	NCI	$158,825
1	F32	CA228326	2019	NCI	NCI	$65,340
1	R01	CA236226	2019	NCI	NCI	$477,643
1	R01	CA239660	2019	NCI	NCI	$441,325
5	R37	CA215436	2019	NCI	NCI	$357,238
5	R33	CA212968	2019	NCI	NCI	$291,684
5	K22	CA218459	2018	NCI	NCI	$186,840
3	R01	CA210440	2018	NCI	NCI	$125,000
1	K99	CA226679	2018	NCI	NCI	$98,371
1	R01	CA234361	2019	NCI	NCI	$410,835
3	P30	CA030199	2018	NCI		$12,441
3	P30	CA030199	2018	NCI		$9,141
5	P30	CA030199	2018	NCI		$217,412
3	R01	CA204915	2019	NCI	NCI	$119,844
5	R01	CA204915	2019	NCI	NCI	$384,651
1	U54	CA231637	2018	NCI		$2,499,136
1	R01	CA223220	2018	NCI	NCI	$366,875
5	U01	DE025188	2018	NIDCR	NIDCR	$924,971
3	U54	CA210180	2018	NCI		$15,414
5	U54	CA210180	2018	NCI		$287,039
3	R01	CA211657	2019	NCI	NCI	$18,264

5	R01	CA211657	2019	NCI	NCI	$338,725
1	R01	CA225103	2018	NCI	NCI	$603,877
5	R01	CA175803	2018	NCI	NCI	$312,124
1	R56	CA213859	2017	NCI	OD	$299,992
5	R01	CA188017	2017	NCI	NCI	$474,283
5	P30	CA046934	2019	NCI		$90,232
1	ZIA	BC010788	2018	NCI	NCI	$1,065,881
1	U01	CA232563	2018	NCI	NCI	$2,931,750
5	U01	CA214292	2018	NCI	NCI	$685,690
5	P50	CA140388	2018	NCI		$278,245
5	U01	CA198900	2018	NCI	NCI	$470,021
5	U01	CA224013	2019	NCI	NCI	$828,044
1	U54	CA224019	2017	NCI	NCI	$2,309,994
5	F32	CA216996	2019	NCI	NCI	$63,746
3	P30	CA015704	2019	NCI		$3,703
3	P30	CA015704	2019	NCI		$9,135
3	P30	CA015704	2019	NCI		$7,408
3	P30	CA015704	2019	NCI		$2,890
5	P30	CA015704	2019	NCI		$1,001,522
261201500003I-0-26100075-1			2018	NCI	NCI	$19,812,417
3	R01	CA223828	2019	NCI	NCI	$74,623
5	R01	CA223828	2019	NCI	NCI	$568,179
1	ZIA	BC011713	2018	NCI	NCI	$543,730
1	ZIA	BC011771	2018	NCI	NCI	$36,249
5	R01	NS086956	2018	NINDS	NINDS	$346,576
5	R01	CA188323	2018	NCI	NCI	$321,625
1	U54	CA224083	2017	NCI		$425,714
5	F31	CA224809	2018	NCI	NCI	$48,883
1	F31	CA225069	2018	NCI	NCI	$44,524
5	U54	CA217377	2018	NCI		$146,820
5	R01	NS088355	2018	NINDS	NINDS	$651,177
5	R01	NS089868	2018	NINDS	NINDS	$596,770
5	R01	CA207751	2019	NCI	NCI	$338,739
5	U01	CA199315	2018	NCI	NCI	$623,888
5	R01	CA184502	2017	NCI	NCI	$714,297
5	P50	DE026787	2018	NIDCR		$256,986
1	U01	TR002383	2018	NCATS	NCATS	$1,571,990
1	R37	CA233476	2019	NCI	NCI	$306,248
1	P20	CA233216	2018	NCI		$99,600
5	P50	DE026787	2018	NIDCR		$330,446
5	R01	CA193650	2018	NCI	NCI	$593,093
5	P50	DE026787	2018	NIDCR		$222,373
1	R21	CA234665	2019	NCI	NCI	$172,369
5	P20	GM121327	2019	NIGMS		$306,129
5	K99	CA222554	2018	NCI	NCI	$126,295
5	U24	CA210954	2018	NCI	NCI	$973,045
1	R21	CA226317	2018	NCI	NCI	$169,650
1	P50	CA228991	2018	NCI		$367,780
5	R35	CA210057	2018	NCI	NCI	$1,007,233
1	R01	CA227136	2018	NCI	NCI	$454,956
1	I01	BX004221	2019	VA		
5	I01	CX001441	2018	VA		
1	R01	CA224899	2018	NCI	NCI	$382,641
1	R21	DE026892	2018	NIDCR	NIDCR	$247,500
1	ZIA	BC011423	2018	NCI	NCI	$361,353
5	R01	CA196932	2018	NCI	NCI	$387,027
5	P50	CA217685	2018	NCI		$340,355
5	R01	CA188228	2018	NCI	NCI	$682,620
261201500018I-0-26100005-1			2017	NCI	NCI	$508,124
5	P50	CA196516	2018	NCI		$318,715
5	R01	CA089713	2018	NCI	NCI	$788,333
5	U54	CA209997	2018	NCI	NCI	$2,090,840
5	U01	CA217858	2018	NCI	NCI	$1,314,469
1	U54	CA233306	2018	NCI		$1,673,482
1	R21	EB025406	2018	NIBIB	NIBIB	$225,946
3	U24	CA224067	2018	NCI	NCI	$500,000
1	U24	CA224067	2017	NCI	NCI	$1,974,874
1	U01	CA224145	2017	NCI	NCI	$1,497,616
5	R01	CA122959	2019	NCI	NCI	$575,307
5	R01	CA200905	2019	NCI	NCI	$346,144
1	R21	CA223049	2018	NCI	NCI	$208,854
5	R01	CA213912	2018	NCI	NCI	$353,876
2	P50	CA100632	2018	NCI		$215,115
5	F30	CA221345	2018	NCI	NCI	$40,126
1	R01	CA215059	2018	NCI	NCI	$377,437

1	R03	CA216114	2018	NCI	NCI	$97,125
1	R03	CA231766	2018	NCI	NCI	$97,188
5	R01	CA204115	2019	NCI	NCI	$584,722
1	U54	CA224065	2017	NCI		$483,810
2	P50	CA058223	2018	NCI		$293,807
5	R01	CA185509	2017	NCI	NCI	$258,128
1	R21	CA223300	2018	NCI	NCI	$200,637
5	R37	CA217910	2019	NCI	NCI	$366,059
1	P50	CA210964	2018	NCI		$359,336
5	U01	CA199221	2018	NCI	NCI	$136,071
1	U54	CA224083	2017	NCI	NCI	$2,424,750
5	R01	CA160467	2018	NCI	NCI	$372,258
1	ZIA	BC011672	2018	NCI	NCI	$526,625
1	K22	CA229613	2018	NCI	NCI	$147,169
5	P50	CA116201	2018	NCI		$286,502
5	P50	CA174523	2018	NCI		$361,118
1	R43	CA236164	2018	NCI	NCI	$225,030
5	R01	CA187238	2018	NCI	NCI	$332,000
5	R01	CA214017	2018	NCI	NCI	$338,550
5	U01	CA199297	2018	NCI	NCI	$469,536
1	R37	CA230617	2019	NCI	NCI	$420,691
5	U54	CA217297	2018	NCI	NCI	$2,104,443
5	U54	CA209891	2018	NCI		$298,466
1	U54	CA233223	2018	NCI		$1,189,380
5	U01	CA199216	2018	NCI	NCI	$623,508
1	R03	CA235113	2019	NCI	NCI	$79,250
1	R01	CA232591	2019	NCI	NCI	$361,698
5	R01	NS094218	2019	NINDS	NINDS	$342,634
5	P50	CA196516	2018	NCI		$379,122
5	R01	CA215755	2019	NCI	NCI	$599,119
5	R01	CA096899	2018	NCI	NCI	$358,031
1	ZIC	BC010947	2018	NCI	NCI	$304,881
1	ZIA	BC011782	2018	NCI	NCI	$764,220
5	R01	CA213843	2018	NCI	NCI	$408,250
5	R03	DE027433	2019	NIDCR	NIDCR	$155,500
261201700013C-0-0-1			2017	NCI	NCI	$1,999,931
1	K08	CA222620	2018	NCI	NCI	$209,995
5	U01	CA217456	2018	NCI	NCI	$651,395
5	R01	CA207086	2019	NCI	NCI	$359,522
1	P50	CA217694	2018	NCI		$360,663
5	R35	CA210807	2018	NCI	NCI	$918,000
5	P50	DE026787	2018	NIDCR		$256,601
5	R37	CA225191	2019	NCI	NCI	$385,075
5	R01	CA154923	2019	NCI	NCI	$393,941
5	R01	CA215118	2019	NCI	NCI	$373,880
1	R01	CA226926	2018	NCI	NCI	$385,444
1	R01	CA213201	2018	NCI	NCI	$373,257
5	R01	CA214567	2018	NCI	NCI	$603,477
5	U01	CA215798	2018	NCI	NCI	$684,764
5	R21	CA220090	2019	NCI	NCI	$141,222
5	P50	CA136393	2018	NCI		$354,079
5	R01	CA204926	2019	NCI	NCI	$351,691
5	R01	HD086195	2019	NICHD	NICHD	$325,775
1	R01	CA229259	2018	NCI	NCI	$247,340
5	R21	CA223112	2019	NCI	NCI	$193,042
5	R01	CA218287	2018	NCI	NCI	$373,331
5	U01	CA216468	2018	NCI	NCI	$596,334
1	U54	CA233306	2018	NCI		$1,520,403
5	R35	CA210064	2018	NCI	NCI	$1,046,441
2	R01	EB017270	2018	NIBIB	NIBIB	$422,405
3	P30	CA093373	2018	NCI		$11,775
3	P30	CA093373	2018	NCI		$6,250
3	P30	CA093373	2018	NCI		$10,000
3	P30	CA093373	2018	NCI		$3,000
3	P30	CA093373	2018	NCI		$4,474
3	P30	CA093373	2018	NCI		$12,500
5	P30	CA093373	2018	NCI		$128,121
5	R01	CA211223	2019	NCI	NCI	$366,659
5	P01	CA117969	2018	NCI		$230,629
5	U24	CA210979	2018	NCI	NCI	$724,282
2	R01	CA155243	2018	NCI	NCI	$328,680
5	R01	CA200970	2018	NCI	NCI	$535,276
5	U54	CA193419	2018	NCI		$386,722
5	R00	CA201601	2018	NCI	NCI	$248,999
					FY Total	$116,933,930

Supplementary Table 2. Publicly available data used in this study and their accessions and sample sources. All the data can be downloaded from SRA (https://trace.ncbi.nlm.nih.gov/Traces/sra/.) using the unique run accession and experiment accession provided. **a** The definition and nomenclature of samples from which the publicly available data were generated and used in this study. **b.** The list of all the publicly available data used for the viral detection in this study. The accession for the SRA Runs and experiments are provided. The mouse used for PDX in the original experiment is also provided in the sample source column if it is provided by the data. **c** Data used as positive control for our assembly method.

Supplementary 2a. The definition and nomenclature of samples from which the publicly available data were generated and used in this study.

Name	Description
PDX tumor (or PDX)	human tumors transplanted and grew in nude mice
Primary tumor	tumor explants directly obtained from patient without any type of culture or treatment
PDX cell line	cell lines derived from PDX tumor
Primary cell culture	cell lines directly derived from patient tumor explant
Tumor (cancer) cell line	lab cultured cell lines which are either commercially available or cultured by the lab for many generations

Supplementary 2b. The list of all the publicly available data used for the viral detection in this study..

Treatments	Run Accession	Experiment Accession	Sample Source	Sample Source
Breast Cancer PDX	ERR2587301	ERX2603577	BRCA1-deficient Breast Cancer	PDX tumor
	ERR2587302	ERX2603578	BRCA1-deficient Breast Cancer	PDX tumor
	ERR2587303	ERX2603579	BRCA1-deficient Breast Cancer	PDX tumor
	ERR2587304	ERX2603580	BRCA1-deficient Breast Cancer	PDX tumor
	ERR2587305	ERX2603581	BRCA1-deficient Breast Cancer	PDX tumor
	ERR2587306	ERX2603582	BRCA1-deficient Breast Cancer	PDX tumor
	ERR2587307	ERX2603583	BRCA1-deficient Breast Cancer	PDX tumor
	ERR2587308	ERX2603584	BRCA1-deficient Breast Cancer	PDX tumor
	ERR2587309	ERX2603585	BRCA1-deficient Breast Cancer	PDX tumor
	ERR2587310	ERX2603586	BRCA1-deficient Breast Cancer	PDX tumor
	ERR2587311	ERX2603587	BRCA1-deficient Breast Cancer	PDX tumor
	ERR2587312	ERX2603588	BRCA1-deficient Breast Cancer	PDX tumor
	ERR2587313	ERX2603589	BRCA1-deficient Breast Cancer	PDX tumor
	ERR2587314	ERX2603590	BRCA1-deficient Breast Cancer	PDX tumor
	ERR2587315	ERX2603591	BRCA1-deficient Breast Cancer	PDX tumor

	ERR2587316	ERX2603592	BRCA1-deficient Breast Cancer	PDX tumor
	ERR2587317	ERX2603593	BRCA1-deficient Breast Cancer	PDX tumor
	ERR2587318	ERX2603594	BRCA1-deficient Breast Cancer	PDX tumor
	ERR2587319	ERX2603595	BRCA1-deficient Breast Cancer	PDX tumor
	ERR2587320	ERX2603596	BRCA1-deficient Breast Cancer	PDX tumor
	ERR2587321	ERX2603597	BRCA1-deficient Breast Cancer	PDX tumor
	ERR2587322	ERX2603598	BRCA1-deficient Breast Cancer	PDX tumor
	ERR2587323	ERX2603599	BRCA1-deficient Breast Cancer	PDX tumor
	ERR2587324	ERX2603600	BRCA1-deficient Breast Cancer	PDX tumor
	ERR2587325	ERX2603601	BRCA1-deficient Breast Cancer	PDX tumor
	ERR2587326	ERX2603602	BRCA1-deficient Breast Cancer	PDX tumor
	ERR2587327	ERX2603603	BRCA1-deficient Breast Cancer	PDX tumor
	ERR2587328	ERX2603604	BRCA1-deficient Breast Cancer	PDX tumor
	ERR2587329	ERX2603605	BRCA1-deficient Breast Cancer	PDX tumor
	ERR2587330	ERX2603606	BRCA1-deficient Breast Cancer	PDX tumor
	ERR2587331	ERX2603607	BRCA1-deficient Breast Cancer	PDX tumor
	ERR2587332	ERX2603608	BRCA1-deficient Breast Cancer	PDX tumor
	ERR2587333	ERX2603609	BRCA1-deficient Breast Cancer	PDX tumor
	ERR2587334	ERX2603610	BRCA1-deficient Breast Cancer	PDX tumor
	ERR2587335	ERX2603611	BRCA1-deficient Breast Cancer	PDX tumor
	ERR2587336	ERX2603612	BRCA1-deficient Breast Cancer	PDX tumor
	ERR2587337	ERR2587337	BRCA1-deficient Breast Cancer	PDX tumor
	ERR2587338	ERX2603614	BRCA1-deficient Breast Cancer	PDX tumor
	ERR2587339	ERX2603615	BRCA1-deficient Breast Cancer	PDX tumor
	ERR2587340	ERX2603616	BRCA1-deficient Breast Cancer	PDX tumor
	ERR2587341	ERX2603617	BRCA1-deficient Breast Cancer	PDX tumor
	ERR2587342	ERX2603618	BRCA1-deficient Breast Cancer	PDX tumor
	ERR2587343	ERX2603619	BRCA1-deficient Breast Cancer	PDX tumor
	ERR2587344	ERX2603620	BRCA1-deficient Breast Cancer	PDX tumor
	SRR6727448[1]	SRX3700451	Mammary tumor tissue	PDX tumor, NSG mice
	SRR6727449 [1]	SRX3700452	Mammary tumor tissue	PDX tumor, NSG mice
	SRR6727450[1]	SRX3700453	Mammary tumor tissue	PDX tumor, NSG mice
	SRR6727451[1]	SRX3700454	Mammary tumor cells	PDX tumor, NSG mice
	SRR6727452[1]	SRX3700455	Mammary tumor cells	PDX tumor, NSG mice
	SRR6727453[1]	SRR6727453	Mammary tumor cells	PDX tumor, NSG mice
	SRR6727454[1]	SRX3700457	Mammary tumor cells	PDX tumor, NSG mice
	SRR6727455[1]	SRX3700458	Mammary tumor cells	PDX tumor, NSG mice
	SRR6727456[1]	SRX3700459	Mammary tumor cells	PDX tumor, NSG mice
	SRR6727457[1]	SRX3700460	Brain metastasis tissue	PDX tumor, NSG mice
	SRR6727458[1]	SRX3700461	Mammary tumor tissue	PDX tumor, NSG mice
	SRR6727459[1]	SRX3700462	Mammary tumor tissue	PDX tumor, NSG mice
	SRR6727460[1]	SRX3700463	Mammary tumor tissue	PDX tumor, NSG mice
	SRR6727461[1]	SRX3700464	Mammary tumor cells	PDX tumor, NSG mice
	SRR6727462[1]	SRX3700465	Mammary tumor cells	PDX tumor, NSG mice
	SRR6727463[1]	SRX3700466	Mammary tumor cells	PDX tumor, NSG mice
	SRR6727464[1]	SRX3700467	Mammary tumor cells	PDX tumor, NSG mice
	SRR6727465[1]	SRX3700468	Mammary tumor cells	PDX tumor, NSG mice
	SRR6727466[1]	SRX3700469	Mammary tumor cells	PDX tumor, NSG mice
	SRR6727467[1]	SRX3700470	Mammary tumor tissue	PDX tumor, NSG mice
	SRR6727468[1]	SRX3700471	Mammary tumor tissue	PDX tumor, NSG mice
	ERR1084763[2]	ERX1164353	Primary Invasive Ductal Carcinoma	PDX tumor, NSG mice
	ERR1084764[2]	ERX1164354	Primary Invasive Ductal Carcinoma	PDX tumor, NSG mice
	ERR1084765[2]	ERX1164355	Primary Invasive Ductal Carcinoma	PDX tumor, NSG mice
	ERR1084766[2]	ERX1164356	Primary Invasive Ductal Carcinoma	PDX tumor, NSG mice
	ERR1084767[2]	ERX1164357	Primary Ductal Carcinoma	PDX tumor, XID mice
	ERR1084768[2]	ERX1164358	Primary Invasive Ductal Carcinoma	PDX tumor, NSG mice
	ERR1084775[2]	ERX1164365	Circulating tumor cells	PDX tumor, NSG mice
	ERR1084798[2]	ERX1164388	Primary Invasive Ductal Carcinoma	PDX tumor, Athymic nude mice
	ERR1084799[2]	ERX1164389	Primary Invasive Ductal Carcinoma	PDX tumor, Athymic nude mice
	ERR1084800[2]	ERX1164390	Primary Invasive Ductal Carcinoma	PDX tumor, Athymic nudemice
	ERR1084801[2]	ERX1164391	Primary Invasive Ductal Carcinoma	PDX tumor, Athymic nudemice
	ERR1084802[2]	ERX1164392	Peritoneum Met Invasive Lobular Carcinoma	PDX tumor, Athymic nude mice
	ERR1084803[2]	ERX1164393	Axi lymph Met Invasive Ductal Carcinoma	PDX tumor, Athymic nude mice
	ERR1084804[2]	ERX1164394	Primary Ductal Carcinoma	PDX tumor, Athymic nude mice
	ERR1084805[2]	ERX1164395	Primary Invasive Ductal Carcinoma	PDX tumor, Athymic nude mice
	ERR1084806[2]	ERX1164396	Primary Invasive Ductal Carcinoma	PDX tumor, Athymic nude mice
	ERR1084807[2]	ERX1164397	Primary Invasive Ductal Carcinoma	PDX tumor, Athymic nude mice
	ERR1084808[2]	ERX1164398	Ovary Met Invasive Lobular Carcinoma	PDX tumor
	ERR1084809[2]	ERX1164399	Primary Invasive Ductal Carcinoma	PDX tumor, Athymic nude mice
	ERR1084810[2]	ERX1164400	Primary Breast cancer	PDX tumor, CB17 SCID mice
	ERR1084811[2]	ERX1164401	Primary Invasive Ductal Carcinoma	PDX tumor, NSG mice

Breast Cancer Control	ERR1982237	ERX2042643	BT20 cell lines	Tumor cell line
	ERR1982238	ERX2042643	BT20 cell lines	Tumor cell line
	ERR1982239	ERX2042644	BT474 cell lines	Tumor cell line
	ERR1982240	ERX2042644	BT474 cell lines	Tumor cell line
	ERR1982241	ERX2042645	BT549 cell lines	Tumor cell line
	ERR1982242	ERX2042645	BT549 cell lines	Tumor cell line
	ERR1982243	ERX2042646	CAL120 cell lines	Tumor cell line
	ERR1982244	ERX2042646	CAL120 cell lines	Tumor cell line
	ERR1982245	ERX2042647	CAL148 cell lines	Tumor cell line
	ERR1982246	ERX2042647	CAL148 cell lines	Tumor cell line
	ERR1982247	ERX2042648	CAL51 cell lines	Tumor cell line
	ERR1982248	ERX2042648	CAL51 cell lines	Tumor cell line
	ERR1982249	ERX2042649	CAMA1 cell lines	Tumor cell line
	ERR1982250	ERX2042649	CAMA1 cell lines	Tumor cell line
	ERR1982251	ERX2042650	HCC1187 cell lines	Tumor cell line
	ERR1982252	ERX2042650	HCC1187 cell lines	Tumor cell line
	ERR1982253	ERX2042651	HCC1395 cell lines	Tumor cell line
	ERR1982254	ERX2042651	HCC1395 cell lines	Tumor cell line
	ERR1982255	ERX2042652	HCC1419 cell lines	Tumor cell line
	ERR1982256	ERX2042652	HCC1419 cell lines	Tumor cell line
	ERR1982257	ERX2042653	HCC1500 cell lines	Tumor cell line
	ERR1982258	ERX2042653	HCC1500 cell lines	Tumor cell line
	ERR1982259	ERX2042654	HCC1569 cell lines	Tumor cell line
	ERR1982260	ERX2042654	HCC1569 cell lines	Tumor cell line
	ERR1982261	ERX2042655	HCC1806 cell lines	Tumor cell line
	ERR1982262	ERX2042655	HCC1806 cell lines	Tumor cell line
	ERR1982263	ERX2042656	HCC1937 cell lines	Tumor cell line
	ERR1982264	ERX2042656	HCC1937 cell lines	Tumor cell line
	ERR1982265	ERX2042657	HCC1954 cell lines	Tumor cell line
	ERR1982266	ERX2042657	HCC1954 cell lines	Tumor cell line
	ERR1982267	ERX2042658	HCC38 cell lines	Tumor cell line
	ERR1982268	ERX2042658	HCC38 cell lines	Tumor cell line
	ERR1982269	ERX2042659	HCC70 cell lines	Tumor cell line
	ERR1982270	ERX2042659	HCC70 cell lines	Tumor cell line
	ERR1982271	ERX2042660	HS578T cell lines	Tumor cell line
	ERR1982272	ERX2042660	HS578T cell lines	Tumor cell line
	ERR1982273	ERX2042661	MCF7 cell lines	Tumor cell line
	ERR1982274	ERX2042661	MCF7 cell lines	Tumor cell line
	ERR1982275	ERX2042662	MFM223 cell lines	Tumor cell line
	ERR1982276	ERX2042662	MFM223 cell lines	Tumor cell line
	ERR1982277	ERX2042663	MM157 cell lines	Tumor cell line
	ERR1982278	ERX2042663	MM157 cell lines	Tumor cell line
	ERR1982279	ERX2042664	MM231 cell lines	Tumor cell line
	ERR1982280	ERX2042664	MM231 cell lines	Tumor cell line
	ERR1982281	ERX2042665	MM361 cell lines	Tumor cell line
	ERR1982282	ERX2042665	MM361 cell lines	Tumor cell line
	ERR1982283	ERX2042666	MM436 cell lines	Tumor cell line
	ERR1982284	ERX2042666	MM436 cell lines	Tumor cell line
	ERR1982285	ERX2042667	MM453 cell lines	Tumor cell line
	ERR1982286	ERX2042667	MM453 cell lines	Tumor cell line
	ERR1982287	ERX2042668	MM468 cell lines	Tumor cell line
	ERR1982288	ERX2042668	MM468 cell lines	Tumor cell line
	ERR1982289	ERX2042669	SKBR3 cell lines	Tumor cell line
	ERR1982290	ERX2042669	SKBR3 cell lines	Tumor cell line
	ERR1982291	ERX2042670	SKBR7 cell lines	Tumor cell line
	ERR1982292	ERX2042670	SKBR7 cell lines	Tumor cell line
	ERR1982293	ERX2042671	T47D cell lines	Tumor cell line
	ERR1982294	ERX2042671	T47D cell lines	Tumor cell line
	ERR1982295	ERX2042672	ZR7530 cell lines	Tumor cell line
	ERR1982296	ERX2042672	ZR7530 cell line	Tumor cell line
	SRR1313062[3]	SRX559713	Breast Cancer Cell Line	Tumor cell line
	SRR1313063[3]	SRX559714	Breast Cancer Cell Line	Tumor cell line
	SRR1313064[3]	SRX559715	Breast Cancer Cell Line	Tumor cell line
	SRR1313066[3]	SRX559717	Breast Cancer Cell Line	Tumor cell line
	SRR1313067[3]	SRX559718	Breast Cancer Cell Line	Tumor cell line
	SRR1313068[3]	SRX559719	Breast Cancer Cell Line	Tumor cell line
	SRR1313069[3]	SRX559720	Breast Cancer Cell Line	Tumor cell line
	SRR1313070[3]	SRX559721	Breast Cancer Cell Line	Tumor cell line
	SRR1313071[3]	SRX559722	Breast Cancer Cell Line	Tumor cell line
	SRR1313072[3]	SRX559723	Breast Cancer Cell Line	Tumor cell line
	SRR1313073[3]	SRX559724	Breast Cancer Cell Line	Tumor cell line

	SRR1313074[3]	SRX559725	Breast Cancer Cell Line	Tumor cell line
	SRR1313075[3]	SRX559726	Breast Cancer Cell Line	Tumor cell line
	SRR1313076[3]	SRX559727	Breast Cancer Cell Line	Tumor cell line
	SRR1313077[3]	SRX559728	Breast Cancer Cell Line	Tumor cell line
	SRR1313078[3]	SRX559729	Breast Cancer Cell Line	Tumor cell line
	SRR1313079[3]	SRX559730	Breast Cancer Cell Line	Tumor cell line
	SRR1313080[3]	SRX559731	Breast Cancer Cell Line	Tumor cell line
	SRR1313081[3]	SRX559732	Breast Cancer Cell Line	Tumor cell line
	SRR1313082 [3]	SRX559733	Breast Cancer Cell Line	Tumor cell line
	SRR1313083[3]	SRX559734	Breast Cancer Cell Line	Tumor cell line
	SRR1313084[3]	SRX559735	Breast Cancer Cell Line	Tumor cell line
	SRR1313085[3]	SRX559736	Breast Cancer Cell Line	Tumor cell line
	SRR1313086[3]	SRX559737	Breast Cancer Cell Line	Tumor cell line
	SRR1313087[3]	SRX559738	Breast Cancer Cell Line	Tumor cell line
	SRR1313088[3]	SRX559739	Breast Cancer Cell Line	Tumor cell line
	SRR1313089[3]	SRX559740	Breast Cancer Cell Line	Tumor cell line
	SRR5364118[4,5]	SRX2659563	MDA MB-361 Cell Line	Tumor cell line
**Glioblastoma PDX**	SRR4427234[6]	SRX2248852	Primary Patient Derived Tumor	PDX tumor
	SRR4427235[6]	SRX2248853	Primary Patient Derived Tumor	PDX tumor
	SRR4427236[6]	SRX2248854	Primary Patient Derived Tumor	PDX tumor
	SRR4427237[6]	SRX2248855	Primary Patient Derived Tumor	PDX tumor
	SRR4427238[6]	SRX2248856	Primary Patient Derived Tumor	PDX tumor
**GBM PDX derived cell cultures**	SRR5114302	SRX2426315	Glioma stem cells	PDX cell line with 2 to 3 passages
	SRR5114303	SRX2426316	Glioma stem cells	PDX cell line with 2 to 3 passages
	SRR5114304	SRX2426317	Glioma stem cells	PDX cell line with 2 to 3 passages
	SRR5114305	SRX2426318	Glioma stem cells	PDX cell line with 2 to 3 passages
	SRR5114306	SRX2426319	Glioma stem cells	PDX cell line with 2 to 3 passages
	SRR5114307	SRX2426320	Glioma stem cells	PDX cell line with 2 to 3 passages
	SRR5114308	SRX2426321	Glioma stem cells	PDX cell line with 2 to 3 passages
	SRR5114309	SRX2426322	Glioma stem cells	PDX cell line with 2 to 3 passages
	SRR5114310	SRX2426323	Glioma stem cells	PDX cell line with 2 to 3 passages
	SRR5114311	SRX2426324	Glioma stem cells	PDX cell line with 2 to 3 passages
	SRR5114312	SRX2426325	Glioma stem cells	PDX cell line with 2 to 3 passages
	SRR5114313	SRX2426326	Glioma stem cells	PDX cell line with 2 to 3 passages
	SRR5114316	SRX2426329	Glioma stem cells	PDX cell line with 2 to 3 passages
	SRR5114317	SRX2426330	Glioma stem cells	PDX cell line with 2 to 3 passages
	SRR5114318	SRX2426331	Glioma stem cells	PDX cell line with 2 to 3 passages
	SRR5114314	SRX2426327	Glioma stem cells	PDX cell line with 11 to 20 passages
	SRR5114315	SRX2426328	Glioma stem cells	PDX cell line with 11 to 20 passages
	SRR5114320	SRX2426333	Glioma stem cells	PDX cell line with 11 to 20 passages
**GBM Control**	SRR3356059[7]	SRX1689920	Glioblastoma cultured cells	Tumor cell line
	SRR3356060[7]	SRX1689920	Glioblastoma cultured cells	Tumor cell line
	SRR3356061[7]	SRX1689920	Glioblastoma cultured cells	Tumor cell line
	SRR3356062[7]	SRX1689920	Glioblastoma cultured cells	Tumor cell line
	SRR3356063[7]	SRX1689921	Glioblastoma cultured cells	Tumor cell line
	SRR3356064[7]	SRX1689921	Glioblastoma cultured cells	Tumor cell line
	SRR3356071[7]	SRX1689924	T98G Glioblastoma cultured cells	Tumor cell line
	SRR3356072[7]	SRX1689924	T98G Glioblastoma cultured cells	Tumor cell line
	SRR3356073[7]	SRX1689924	T98G Glioblastoma cultured cells	Tumor cell line
	SRR3356074[7]	SRX1689924	T98G Glioblastoma cultured cells	Tumor cell line
	SRR3356075[7]	SRX1689925	T98G Glioblastoma cultured cells	Tumor cell line
	SRR3356076[7]	SRX1689925	T98G Glioblastoma cultured cells	Tumor cell line
	SRR3356077[7]	SRX1689925	T98G Glioblastoma cultured cells	Tumor cell line
	SRR3356078[7]	SRX1689925	T98G Glioblastoma cultured cells	Tumor cell line
	SRR4241104[8]	SRX2162078	Flash Frozen GBM specimens	Primary tumor
	SRR4241106[8]	SRX2162080	Flash Frozen GBM specimens	Primary tumor
	SRR4241110[8]	SRX2162084	Flash Frozen GBM specimens	Primary tumor
	SRR4343486[9]	SRX2210471	Primary GBM stem cell cultures	Primary cell culture
	SRR5289362[10]	SRX2592104	Human Glioblastoma stem cell	Primary cell culture
	SRR5635314	SRX2875070	GBM brain tissue	Primary tumor
	SRR5635315	SRX2875071	GBM brain tissue	Primary tumor
	SRR5635317	SRX2875072	GBM brain tissue	Primary tumor
	SRR6371761[11]	SRX3467084	Primary cell line-Brain tumor	Primary cell culture
	SRR6371762[11]	SRX3467085	Primary cell line-Brain tumor	Primary cell culture
	SRR6371763[11]	SRX3467086	Primary cell line-Brain tumor	Primary cell culture
**Lung Cancer PDX (Conventional sequencing)**	ERR1084776[2]	ERX1164366	Primary Lung Squamous	PDX tumor
	ERR1084777[2]	ERX1164367	Primary Lung Squamous	PDX tumor
	ERR1084778[2]	ERX1164368	Primary Small cell lung carcinoma	PDX tumor
	ERR1084779[2]	ERX1164369	Primary Small cell lung carcinoma	PDX tumor
	ERR1084780[2]	ERX1164370	Primary Small cell lung carcinoma	PDX tumor
	ERR1084784[2]	ERX1164374	Lung Squamous	PDX tumor

	ERR1084785[2]	ERX1164375	Lung Squamous	PDX tumor
	ERR1084786[2]	ERX1164376	Lung Adenocarcinoma	PDX tumor
	ERR1084787[2]	ERX1164377	Lung Squamous	PDX tumor
	ERR1084788[2]	ERX1164378	Lung Squamous	PDX tumor
	ERR1084789[2]	ERX1164379	Lung Adenocarcinoma	PDX tumor
	ERR1084790[2]	ERX1164380	Lung Squamous	PDX tumor
	ERR1084791[2]	ERX1164381	Lung Adenocarcinoma	PDX tumor
	ERR1084792[2]	ERX1164382	Lung Squamous	PDX tumor
	ERR1084793[2]	ERX1164383	Lung Adenocarcinoma	PDX tumor
	ERR1084794[2]	ERX1164384	Lung Squamous	PDX tumor
	ERR1084795[2]	ERX1164385	Lung Squamous	PDX tumor
	ERR1084796[2]	ERX1164386	Lung Squamous	PDX tumor
	ERR1084797[2]	ERX1164387	Lung Squamous	PDX tumor
	ERR1084815[2]	ERX1164405	PrimaryLung Sarcomatoid	PDX tumor, CB17 SCID mice
	ERR1084816[2]	ERX1164406	Primary Lung Bronchogenic	PDX tumor, CB17 SCID mice
	ERR1084817[2]	ERX1164407	Primary Lung Adenocarcinoma	PDX tumor, CB17 SCID mice
	ERR1084818[2]	ERX1164408	Primary (met: HLXF036LN) Lung Adenocarcinoma	PDX tumor, CB17 SCID mice
	ERR1084819[2]	ERX1164409	Axi lymph Met Lung Adenocarcinoma	PDX tumor, CB17 SCID mice
	ERR1084820[2]	ERX1164410	Primary Lung Squamous	PDX tumor
	ERR1084821[2]	ERX1164411	Primary Lung Adenocarcinoma	PDX tumor, CB17 SCID mice
	ERR1084822[2]	ERX1164412	Primary Lung Squamous	PDX tumor, NSG mice
	ERR1084831[2]	ERX1164421	Primary Lung Squamous	PDX tumor, Athymic nude mice
	ERR1084832[2]	ERX1164422	Adrenal Met Lung Adenocarcinoma	PDX tumor, Athymic nude mice
	ERR1084833[2]	ERX1164423	Skin Met Lung Squamous	PDX tumor, Athymic nude mice
	ERR1084834[2]	ERX1164424	Primary Lung Squamous	PDX tumor, Athymic nude mice
	ERR1084835[2]	ERX1164425	Brain Met Lung Adenocarcinoma	PDX tumor, Athymic nude mice
	ERR1084836[2]	ERX1164426	Primary Lung Adenocarcinomamice	PDX tumor, Athymic nude mice
	ERR1084837[2]	ERX1164427	Primary Lung Adenocarcinoma	PDX tumor, NSG mice
	ERR1084838[2]	ERX1164428	Primary Lung Adenocarcinoma	PDX tumor, NSG mice
	ERR1084839[2]	ERX1164429	Primary (met: IC14LC18) Lung Adenocarcinoma	PDX tumor, Athymic nude mice
	ERR1084843[2]	ERX1164433	Skin Met Lung Squamous	PDX tumor, Athymic nude mice
	SRR2103637[12]	SRX1098160	lung adenocarcinoma	PDX tumor
**Lung Cancer PDX (Single-cell sequencing)**	SRR2049480[12]	SRX1047560	LC-MBT-15	PDX tumor/ cell culture
	SRR2049481[12]	SRX1047561	LC-MBT-15	PDX tumor/ cell culture
	SRR2049482[12]	SRX1047562	LC-MBT-15	PDX tumor/ cell culture
	SRR2049483[12]	SRX1047563	LC-MBT-15	PDX tumor/ cell culture
	SRR2049484[12]	SRX1047564	LC-MBT-15	PDX tumor/ cell culture
	SRR2049485[12]	SRX1047565	LC-MBT-15	PDX tumor/ cell culture
	SRR2049486[12]	SRX1047566	LC-MBT-15	PDX tumor/ cell culture
	SRR2049487[12]	SRX1047567	LC-MBT-15	PDX tumor/ cell culture
	SRR2049488[12]	SRX1047568	LC-MBT-15	PDX tumor/ cell culture
	SRR2049489[12]	SRX1047569	LC-MBT-15	PDX tumor/ cell culture
	SRR2049490[12]	SRX1047570	LC-MBT-15	PDX tumor/ cell culture
	SRR2049491[12]	SRX1047571	LC-MBT-15	PDX tumor/ cell culture
	SRR2049492[12]	SRX1047572	LC-MBT-15	PDX tumor/ cell culture
	SRR2049493[12]	SRX1047573	LC-MBT-15	PDX tumor/ cell culture
	SRR2049494[12]	SRX1047574	LC-MBT-15	PDX tumor/ cell culture
	SRR2049495[12]	SRX1047575	LC-MBT-15	PDX tumor/ cell culture
	SRR2049496[12]	SRX1047576	LC-MBT-15	PDX tumor/ cell culture
	SRR2049497[12]	SRX1047577	LC-MBT-15	PDX tumor/ cell culture
	SRR2049498[12]	SRX1047578	LC-MBT-15	PDX tumor/ cell culture
	SRR2049499[12]	SRX1047579	LC-MBT-15	PDX tumor/ cell culture
	SRR2049500[12]	SRX1047580	LC-MBT-15	PDX tumor/ cell culture
	SRR2049501[12]	SRX1047581	LC-MBT-15	PDX tumor/ cell culture
	SRR2049502[12]	SRX1047582	LC-MBT-15	PDX tumor/ cell culture
	SRR2049503[12]	SRX1047583	LC-MBT-15	PDX tumor/ cell culture
	SRR2049504[12]	SRX1047584	LC-MBT-15	PDX tumor/ cell culture
	SRR2049505[12]	SRX1047585	LC-MBT-15	PDX tumor/ cell culture
	SRR2049506[12]	SRX1047586	LC-MBT-15	PDX tumor/ cell culture
	SRR2049507[12]	SRX1047587	LC-MBT-15	PDX tumor/ cell culture
	SRR2049508[12]	SRX1047588	LC-MBT-15	PDX tumor/ cell culture
	SRR2049509[12]	SRX1047589	LC-MBT-15	PDX tumor/ cell culture
	SRR2049510[12]	SRX1047590	LC-MBT-15	PDX tumor/ cell culture
	SRR2049511[12]	SRX1047591	LC-MBT-15	PDX tumor/ cell culture
	SRR2049512[12]	SRX1047592	LC-MBT-15	PDX tumor/ cell culture
	SRR2049513[12]	SRX1047593	LC-MBT-15	PDX tumor/ cell culture
	SRR2049514[12]	SRX1047594	LC-MBT-15	PDX tumor/ cell culture
	SRR2049515[12]	SRX1047595	LC-MBT-15	PDX tumor/ cell culture
	SRR2049516[12]	SRX1047596	LC-MBT-15	PDX tumor/ cell culture
	SRR2049517[12]	SRX1047597	LC-MBT-15	PDX tumor/ cell culture

	SRR2049518[12]	SRX1047598	LC-MBT-15	PDX tumor/ cell culture
	SRR2049519[12]	SRX1047599	LC-MBT-15	PDX tumor/ cell culture
	SRR2049520[12]	SRX1047600	LC-MBT-15	PDX tumor/ cell culture
	SRR2049521[12]	SRX1047601	LC-MBT-15	PDX tumor/ cell culture
	SRR2049522[12]	SRX1047602	LC-MBT-15	PDX tumor/ cell culture
	SRR2049523[12]	SRX1047603	LC-MBT-15	PDX tumor/ cell culture
	SRR2049524[12]	SRX1047604	LC-MBT-15	PDX tumor/ cell culture
	SRR2049525[12]	SRX1047605	LC-MBT-15	PDX tumor/ cell culture
	SRR2049526[12]	SRX1047606	LC-MBT-15	PDX tumor/ cell culture
	SRR2049527[12]	SRX1047607	LC-MBT-15	PDX tumor/ cell culture
	SRR2049528[12]	SRX1047608	LC-MBT-15	PDX tumor/ cell culture
	SRR2049529[12]	SRX1047609	LC-MBT-15	PDX tumor/ cell culture
	SRR2049530[12]	SRX1047610	LC-MBT-15	PDX tumor/ cell culture
	SRR2049531[12]	SRX1047611	LC-MBT-15	PDX tumor/ cell culture
	SRR2049532[12]	SRX1047612	LC-MBT-15	PDX tumor/ cell culture
	SRR2049533[12]	SRX1047613	LC-MBT-15	PDX tumor/ cell culture
	SRR2049534[12]	SRX1047614	LC-MBT-15	PDX tumor/ cell culture
	SRR2049535[12]	SRX1047615	LC-MBT-15	PDX tumor/ cell culture
	SRR2049390[12]	SRX1047470	LC-PT-45	PDX tumor/ cell culture
	SRR2049391[12]	SRX1047471	LC-PT-45	PDX tumor/ cell culture
	SRR2049392[12]	SRX1047472	LC-PT-45	PDX tumor/ cell culture
	SRR2049393[12]	SRX1047473	LC-PT-45	PDX tumor/ cell culture
	SRR2049394[12]	SRX1047474	LC-PT-45	PDX tumor/ cell culture
	SRR2049396[12]	SRX1047476	LC-PT-45	PDX tumor/ cell culture
	SRR2049397[12]	SRX1047477	LC-PT-45	PDX tumor/ cell culture
	SRR2049398[12]	SRX1047478	LC-PT-45	PDX tumor/ cell culture
	SRR2049399[12]	SRX1047479	LC-PT-45	PDX tumor/ cell culture
	SRR2049400[12]	SRX1047480	LC-PT-45	PDX tumor/ cell culture
	SRR2049401[12]	SRX1047481	LC-PT-45	PDX tumor/ cell culture
	SRR2049402[12]	SRX1047482	LC-PT-45	PDX tumor/ cell culture
	SRR2049403[12]	SRX1047483	LC-PT-45	PDX tumor/ cell culture
	SRR2049404[12]	SRX1047484	LC-PT-45	PDX tumor/ cell culture
	SRR2049405[12]	SRX1047485	LC-PT-45	PDX tumor/ cell culture
	SRR2049406[12]	SRX1047486	LC-PT-45	PDX tumor/ cell culture
	SRR2049407[12]	SRX1047487	LC-PT-45	PDX tumor/ cell culture
	SRR2049408[12]	SRX1047488	LC-PT-45	PDX tumor/ cell culture
	SRR2049409[12]	SRX1047489	LC-PT-45	PDX tumor/ cell culture
	SRR2049410[12]	SRX1047490	LC-PT-45	PDX tumor/ cell culture
	SRR2049411[12]	SRX1047491	LC-PT-45	PDX tumor/ cell culture
	SRR2049412[12]	SRX1047492	LC-PT-45	PDX tumor/ cell culture
	SRR2049413[12]	SRX1047493	LC-PT-45	PDX tumor/ cell culture
	SRR2049414[12]	SRX1047494	LC-PT-45	PDX tumor/ cell culture
	SRR2049415[12]	SRX1047495	LC-PT-45	PDX tumor/ cell culture
	SRR2049416[12]	SRX1047496	LC-PT-45	PDX tumor/ cell culture
	SRR2049417[12]	SRX1047497	LC-PT-45	PDX tumor/ cell culture
	SRR2049418[12]	SRX1047498	LC-PT-45	PDX tumor/ cell culture
	SRR2049419[12]	SRX1047499	LC-PT-45	PDX tumor/ cell culture
	SRR2049420[12]	SRX1047500	LC-PT-45	PDX tumor/ cell culture
	SRR2049421[12]	SRX1047501	LC-PT-45	PDX tumor/ cell culture
	SRR2049422[12]	SRX1047502	LC-PT-45	PDX tumor/ cell culture
	SRR2049423[12]	SRX1047503	LC-PT-45	PDX tumor/ cell culture
	SRR2049424[12]	SRX1047504	LC-PT-45	PDX tumor/ cell culture
	SRR2049425[12]	SRX1047505	LC-PT-45	PDX tumor/ cell culture
	SRR2049426[12]	SRX1047506	LC-PT-45	PDX tumor/ cell culture
	SRR2049427[12]	SRX1047507	LC-PT-45	PDX tumor/ cell culture
	SRR2049428[12]	SRX1047508	LC-PT-45	PDX tumor/ cell culture
	SRR2049429[12]	SRX1047509	LC-PT-45	PDX tumor/ cell culture
	SRR2049430[12]	SRX1047510	LC-PT-45	PDX tumor/ cell culture
	SRR2049431[12]	SRX1047511	LC-PT-45	PDX tumor/ cell culture
	SRR2049432[12]	SRX1047512	LC-PT-45	PDX tumor/ cell culture
	SRR2049433[12]	SRX1047513	LC-PT-45	PDX tumor/ cell culture
	SRR2049434[12]	SRX1047514	LC-PT-45	PDX tumor/ cell culture
	SRR2049435[12]	SRX1047515	LC-PT-45	PDX tumor/ cell culture
	SRR2049436[12]	SRX1047516	LC-PT-45	PDX tumor/ cell culture
	SRR2049437[12]	SRX1047517	LC-PT-45	PDX tumor/ cell culture
	SRR2049438[12]	SRX1047518	LC-PT-45	PDX tumor/ cell culture
	SRR2049439[12]	SRX1047519	LC-PT-45	PDX tumor/ cell culture
	SRR2049440[12]	SRX1047520	LC-PT-45	PDX tumor/ cell culture
	SRR2049441[12]	SRX1047521	LC-PT-45	PDX tumor/ cell culture
	SRR2049442[12]	SRX1047522	LC-PT-45	PDX tumor/ cell culture
	SRR2049443[12]	SRX1047523	LC-PT-45	PDX tumor/ cell culture

	SRR2049444[12]	SRX1047524	LC-PT-45	PDX tumor/ cell culture
	SRR2049445[12]	SRX1047525	LC-PT-45	PDX tumor/ cell culture
	SRR2049446[12]	SRX1047526	LC-PT-45	PDX tumor/ cell culture
	SRR2049447[12]	SRX1047527	LC-PT-45	PDX tumor/ cell culture
	SRR2049448[12]	SRX1047528	LC-PT-45	PDX tumor/ cell culture
	SRR2049449[12]	SRX1047529	LC-PT-45	PDX tumor/ cell culture
	SRR2049450[12]	SRX1047530	LC-PT-45	PDX tumor/ cell culture
	SRR2049451[12]	SRX1047531	LC-PT-45	PDX tumor/ cell culture
	SRR2049452[12]	SRX1047532	LC-PT-45	PDX tumor/ cell culture
	SRR2049453[12]	SRX1047533	LC-PT-45	PDX tumor/ cell culture
	SRR2049454[12]	SRX1047534	LC-PT-45	PDX tumor/ cell culture
	SRR2049455[12]	SRX1047535	LC-PT-45	PDX tumor/ cell culture
	SRR2049456[12]	SRX1047536	LC-PT-45	PDX tumor/ cell culture
	SRR2049457[12]	SRX1047537	LC-PT-45	PDX tumor/ cell culture
	SRR2049458[12]	SRX1047538	LC-PT-45	PDX tumor/ cell culture
	SRR2049459[12]	SRX1047539	LC-PT-45	PDX tumor/ cell culture
	SRR2049460[12]	SRX1047540	LC-PT-45	PDX tumor/ cell culture
	SRR2049461[12]	SRX1047541	LC-PT-45	PDX tumor/ cell culture
	SRR2049462[12]	SRX1047542	LC-PT-45	PDX tumor/ cell culture
	SRR2049463[12]	SRX1047543	LC-PT-45	PDX tumor/ cell culture
	SRR2049464[12]	SRX1047544	LC-PT-45	PDX tumor/ cell culture
	SRR2049465[12]	SRX1047545	LC-PT-45	PDX tumor/ cell culture
	SRR2049466[12]	SRX1047546	LC-PT-45	PDX tumor/ cell culture
	SRR2049467[12]	SRX1047547	LC-PT-45	PDX tumor/ cell culture
	SRR2049468[12]	SRX1047548	LC-PT-45	PDX tumor/ cell culture
	SRR2049469[12]	SRX1047549	LC-PT-45	PDX tumor/ cell culture
	SRR2049470[12]	SRX1047550	LC-PT-45	PDX tumor/ cell culture
	SRR2049471[12]	SRX1047551	LC-PT-45	PDX tumor/ cell culture
	SRR2049472[12]	SRX1047552	LC-PT-45	PDX tumor/ cell culture
	SRR2049473[12]	SRX1047553	LC-PT-45	PDX tumor/ cell culture
	SRR2049474[12]	SRX1047554	LC-PT-45	PDX tumor/ cell culture
	SRR2049475[12]	SRX1047555	LC-PT-45	PDX tumor/ cell culture
	SRR2049476[12]	SRX1047556	LC-PT-45	PDX tumor/ cell culture
	SRR2049477[12]	SRX1047557	LC-PT-45	PDX tumor/ cell culture
	SRR2049478[12]	SRX1047558	LC-PT-45	PDX tumor/ cell culture
	SRR2049479[12]	SRX1047559	LC-PT-45	PDX tumor/ cell culture
	SRR2049536[12]	SRX1047616	LC-PT-45	PDX tumor/ cell culture
	SRR2049539[12]	SRX1047619	LC-PT-45	PDX tumor/ cell culture
**Lung Cancer Control (Conventional sequencing)**	SRR2103638	SRX1098161	lung adenocarcinoma	Primary tumor
**Lung Cancer Control (Single-cell sequencing)**	SRR2049395[12]	SRX1047475	LC-PT-45	Primary tumor
	SRR2049339[12]	SRX1047419	Human lung cancer cell line H358	Tumor cell line
	SRR2049340[12]	SRX1047420	Human lung cancer cell line H358	Tumor cell line
	SRR2049341[12]	SRX1047421	Human lung cancer cell line H358	Tumor cell line
	SRR2049342[12]	SRX1047422	Human lung cancer cell line H358	Tumor cell line
	SRR2049343[12]	SRX1047423	Human lung cancer cell line H358	Tumor cell line
	SRR2049344[12]	SRX1047424	Human lung cancer cell line H358	Tumor cell line
	SRR2049345[12]	SRX1047425	Human lung cancer cell line H358	Tumor cell line
	SRR2049346[12]	SRX1047426	Human lung cancer cell line H358	Tumor cell line
	SRR2049347[12]	SRX1047427	Human lung cancer cell line H358	Tumor cell line
	SRR2049348[12]	SRX1047428	Human lung cancer cell line H358	Tumor cell line
	SRR2049349[12]	SRX1047429	Human lung cancer cell line H358	Tumor cell line
	SRR2049350[12]	SRX1047430	Human lung cancer cell line H358	Tumor cell line
	SRR2049351[12]	SRX1047431	Human lung cancer cell line H358	Tumor cell line
	SRR2049352[12]	SRX1047432	Human lung cancer cell line H358	Tumor cell line
	SRR2049353[12]	SRX1047433	Human lung cancer cell line H358	Tumor cell line
	SRR2049354[12]	SRX1047434	Human lung cancer cell line H358	Tumor cell line
	SRR2049355[12]	SRX1047435	Human lung cancer cell line H358	Tumor cell line
	SRR2049356[12]	SRX1047436	Human lung cancer cell line H358	Tumor cell line
	SRR2049358[12]	SRX1047438	Human lung cancer cell line H358	Tumor cell line
	SRR2049359[12]	SRX1047439	Human lung cancer cell line H358	Tumor cell line
	SRR2049360[12]	SRX1047440	Human lung cancer cell line H358	Tumor cell line
	SRR2049361[12]	SRX1047441	Human lung cancer cell line H358	Tumor cell line
	SRR2049362[12]	SRX1047442	Human lung cancer cell line H358	Tumor cell line
	SRR2049363[12]	SRX1047443	Human lung cancer cell line H358	Tumor cell line
	SRR2049364[12]	SRX1047444	Human lung cancer cell line H358	Tumor cell line
	SRR2049365[12]	SRX1047445	Human lung cancer cell line H358	Tumor cell line
	SRR2049366[12]	SRX1047446	Human lung cancer cell line H358	Tumor cell line
	SRR2049367[12]	SRX1047447	Human lung cancer cell line H358	Tumor cell line

	SRR2049368[12]	SRX1047448	Human lung cancer cell line H358	Tumor cell line
	SRR2049369[12]	SRX1047449	Human lung cancer cell line H358	Tumor cell line
	SRR2049370[12]	SRX1047450	Human lung cancer cell line H358	Tumor cell line
	SRR2049371[12]	SRX1047451	Human lung cancer cell line H358	Tumor cell line
	SRR2049372[12]	SRX1047452	Human lung cancer cell line H358	Tumor cell line
	SRR2049373[12]	SRX1047453	Human lung cancer cell line H358	Tumor cell line
	SRR2049374[12]	SRX1047454	Human lung cancer cell line H358	Tumor cell line
	SRR2049375[12]	SRX1047455	Human lung cancer cell line H358	Tumor cell line
	SRR2049376[12]	SRX1047456	Human lung cancer cell line H358	Tumor cell line
	SRR2049377[12]	SRX1047457	Human lung cancer cell line H358	Tumor cell line
	SRR2049378[12]	SRX1047458	Human lung cancer cell line H358	Tumor cell line
	SRR2049379[12]	SRX1047459	Human lung cancer cell line H358	Tumor cell line
	SRR2049380[12]	SRX1047460	Human lung cancer cell line H358	Tumor cell line
	SRR2049381[12]	SRX1047461	Human lung cancer cell line H358	Tumor cell line
	SRR2049382[12]	SRX1047462	Human lung cancer cell line H358	Tumor cell line
	SRR2049383[12]	SRX1047463	Human lung cancer cell line H358	Tumor cell line
	SRR2049384[12]	SRX1047464	Human lung cancer cell line H358	Tumor cell line
	SRR2049385[12]	SRX1047465	Human lung cancer cell line H358	Tumor cell line
	SRR2049386[12]	SRX1047466	Human lung cancer cell line H358	Tumor cell line
	SRR2049387[12]	SRX1047467	Human lung cancer cell line H358	Tumor cell line
	SRR2049388[12]	SRX1047468	Human lung cancer cell line H358	Tumor cell line
	SRR2049389[12]	SRX1047469	Human lung cancer cell line H358	Tumor cell line
	ERR1084823[2]	ERX1164413	Primary Carcinosarcoma	PDX tumor, CB17 SCID mice
	ERR1084824[2]	ERX1164414	Small intestine Serous carcinoma	PDX tumor, CB17 SCID mice
	ERR1084825[2]	ERX1164415	Primary Endometrioid	PDX tumor, NSG mice
**Ovarian Cancer PDX**	ERR1084826[2]	ERX1164416	Primary Papillary serous carcinoma	PDX tumor, CB17 SCID mice
	ERR1084840[2]	ERX1164430	Primary Ovarian cancer	PDX tumor, NSG mice
	ERR1084841[2]	ERX1164431	Primary Ovarian cancer	PDX tumor, NSG mice
	ERR1084842[2]	ERX1164432	Peritoneum Met Serous carcinoma	PDX tumor, NSG mice
	SRR7204219	SRX4113239	Ovarian Cancer Patient Derived Cell Line	Primary cell culture
	SRR7204220	SRX4113238	Ovarian Cancer Patient Derived Cell Line	Primary cell culture
	SRR7204221	SRX4113237	Ovarian Cancer Patient Derived Cell Line	Primary cell culture
	SRR7204222	SRX4113236	Ovarian Cancer Patient Derived Cell Line	Primary cell culture
	SRR7204223	SRX4113235	Ovarian Cancer Patient Derived Cell Line	Primary cell culture
	SRR7204224	SRX4113234	Ovarian Cancer Patient Derived Cell Line	Primary cell culture
	SRR7204225	SRX4113233	Ovarian Cancer Patient Derived Cell Line	Primary cell culture
	SRR7204226	SRX4113232	Ovarian Cancer Patient Derived Cell Line	Primary cell culture
	SRR7204227	SRX4113231	Ovarian Cancer Patient Derived Cell Line	Primary cell culture
	SRR7204228	SRX4113230	Ovarian Cancer Patient Derived Cell Line	Primary cell culture
**Ovarian Cancer Control**	SRR7204229	SRX4113229	Ovarian Cancer Patient Derived Cell Line	Primary cell culture
	SRR7204230	SRX4113228	Ovarian Cancer Patient Derived Cell Line	Primary cell culture
	SRR7204232	SRX4113226	Ovarian Cancer Patient Derived Cell Line	Primary cell culture
	SRR7204233	SRX4113225	Ovarian Cancer Patient Derived Cell Line	Primary cell culture
	SRR7204234	SRX4113224	Ovarian Cancer Patient Derived Cell Line	Primary cell culture
	SRR7204235	SRX4113223	Ovarian Cancer Patient Derived Cell Line	Primary cell culture
	SRR7204236	SRX4113222	Ovarian Cancer Patient Derived Cell Line	Primary cell culture
	SRR7204237	SRX4113221	Ovarian Cancer Patient Derived Cell Line	Primary cell culture
	SRR7204238	SRX4113220	Ovarian Cancer Patient Derived Cell Line	Primary cell culture
	SRR7204239	SRX4113219	Ovarian Cancer Patient Derived Cell Line	Primary cell culture
	SRR7204240	SRX4113218	Ovarian Cancer Patient Derived Cell Line	Primary cell culture
	SRR7204241	SRX4113217	Ovarian Cancer Patient Derived Cell Line	Primary cell culture
	SRR7204242	SRX4113216	Ovarian Cancer Patient Derived Cell Line	Primary cell culture
	SRR5825451[13]	SRX3003255	BL0269 human bladder cancer	PDX tumor, NSG mice
	SRR5825452[13]	SRX3003256	BL0269 human bladder cancer	PDX tumor, NSG mice
	SRR5825453[13]	SRX3003257	BL0269 human bladder cancer	PDX tumor, NSG mice
	SRR5825454[13]	SRX3003258	BL0269 human bladder cancer	PDX tumor, NSG mice
	SRR5825455[13]	SRX3003259	BL0269 human bladder cancer	PDX tumor, NSG mice
	SRR5825456[13]	SRX3003260	BL0269 human bladder cancer	PDX tumor, NSG mice
	SRR5825457[13]	SRX3003261	BL0269 human bladder cancer	PDX tumor, NSG mice
	SRR5825458[13]	SRX3003262	BL0269 human bladder cancer	PDX tumor, NSG mice
	SRR5825459[13]	SRX3003263	BL0269 human bladder cancer	PDX tumor, NSG mice
**Bladder Cancer PDX**	SRR7412033[14]	SRX4283514	Urothelial Cancer 163C	PDX tumor, NSG mice
	SRR7412034[14]	SRX4283514	Urothelial Cancer 163C	PDX tumor, NSG mice
	SRR7412035[14]	SRX4283515	Urothelial Cancer 163C	PDX tumor, NSG mice
	SRR7412036[14]	SRX4283515	Urothelial Cancer 163C	PDX tumor, NSG mice
	SRR7412037[14]	SRX4283516	Urothelial Cancer 163C	PDX tumor, NSG mice
	SRR7412038[14]	SRX4283516	Urothelial Cancer 163C	PDX tumor, NSG mice
	SRR7412039[14]	SRX4283517	Urothelial Cancer 173C	PDX tumor, NSG mice
	SRR7412040[14]	SRX4283517	Urothelial Cancer 173C	PDX tumor, NSG mice
	SRR7412041[14]	SRX4283518	Urothelial Cancer 173C	PDX tumor, NSG mice
	SRR7412042[14]	SRX4283518	Urothelial Cancer 173C	PDX tumor, NSG mice

	SRR7412043[14]	SRX4283519	Urothelial Cancer 173C	PDX tumor, NSG mice
	SRR7412044[14]	SRX4283519	Urothelial Cancer 173C	PDX tumor, NSG mice
	SRR7412045[14]	SRX4283520	Urothelial Cancer 187C	PDX tumor, NSG mice
	SRR7412046[14]	SRX4283520	Urothelial Cancer 187C	PDX tumor, NSG mice
	SRR7412047[14]	SRX4283521	Urothelial Cancer 187C	PDX tumor, NSG mice
	SRR7412048[14]	SRX4283521	Urothelial Cancer 187C	PDX tumor, NSG mice
	SRR7412049[14]	SRX4283522	Urothelial Cancer 187C	PDX tumor, NSG mice
	SRR7412050[14]	SRX4283522	Urothelial Cancer 187C	PDX tumor, NSG mice
	SRR7412051[14]	SRX4283523	Urothelial Cancer MS	PDX tumor, NSG mice
	SRR7412052[14]	SRX4283524	Urothelial Cancer MS	PDX tumor, NSG mice
	SRR7412053[14]	SRX4283524	Urothelial Cancer MS	PDX tumor, NSG mice
	SRR7412054[14]	SRX4283525	Urothelial Cancer MS	PDX tumor, NSG mice
	SRR7412055[14]	SRX4283525	Urothelial Cancer MS	PDX tumor, NSG mice
	SRR7412056[14]	SRX4283526	Urothelial Cancer WJC	PDX tumor, NSG mice
	SRR7412057[14]	SRX4283526	Urothelial Cancer WJC	PDX tumor, NSG mice
	SRR7412058[14]	SRX4283527	Urothelial Cancer WJC	PDX tumor, NSG mice
	SRR7412059[14]	SRX4283527	Urothelial Cancer WJC	PDX tumor, NSG mice
	SRR7412060[14]	SRX4283528	Urothelial Cancer WJC	PDX tumor, NSG mice
	SRR7412061[14]	SRX4283528	Urothelial Cancer WJC	PDX tumor, NSG mice
**Bladder Cancer Control**	SRR6256328[15]	SRX3362782	Lymph node-positive bladder cancer tissue	Primary tumor
	SRR6256329[15]	SRX3362783	Lymph node-negative bladder cancer tissues	Primary tumor
	SRR6256330[15]	SRX3362784	Lymph node-positive bladder cancer tissue	Primary tumor
	SRR6256331[15]	SRX3362785	Lymph node-negative bladder cancer tissues	Primary tumor
	SRR6256332[15]	SRX3362786	Lymph node-positive bladder cancer tissue	Primary tumor
	SRR6256333[15]	SRX3362787	Lymph node-positive bladder cancer tissue	Primary tumor
	SRR6256334[15]	SRX3362788	Lymph node-positive bladder cancer tissue	Primary tumor
	SRR6256335[15]	SRX3362789	Lymph node-positive bladder cancer tissue	Primary tumor
	SRR6256336[15]	SRX3362790	Lymph node-positive bladder cancer tissue	Primary tumor
	SRR6256337[15]	SRX3362791	Lymph node-positive bladder cancer tissue	Primary tumor
	SRR6256338[15]	SRX3362792	Bladder cancer tissue	Primary tumor
	SRR6256340[15]	SRX3362794	Bladder cancer tissue	Primary tumor
	SRR6256342[15]	SRX3362796	Bladder cancer tissue	Primary tumor
	SRR6256344[15]	SRX3362798	Bladder cancer tissue	Primary tumor
	SRR6256346[15]	SRX3362800	Bladder cancer tissue	Primary tumor
	SRR6349694	SRX3446450	Bladder cancer	Primary tumor
	SRR6349695	SRX3446449	Bladder cancer	Primary tumor
**Colorectal Cancer PDX**	ERR1084769[2]	ERX1164359	Colon Adenocarcinoma	PDX tumor, NSG mice
	ERR1084770[2]	ERX1164360	Colon Adenocarcinoma	PDX tumor, NSG mice
	ERR1084771[2]	ERX1164361	Colon Adenocarcinoma	PDX tumor, NSG mice
	ERR1084772[2]	ERX1164362	Colon Adenocarcinoma	PDX tumor, NSG mice
	ERR1084773[2]	ERX1164363	Colon Adenocarcinoma	PDX tumor, NSG mice
	ERR1084774[2]	ERX1164364	Colorectal cancer	PDX tumor, Nude Mice
	ERR1084812[2]	ERX1164402	Colon Adenocarcinoma	PDX tumor, CB17 SCID
	ERR1084813[2]	ERX1164403	Colon Adenocarcinoma	PDX tumor, CB17 SCID
**Colorectal Cancer Control**	SRR222176	SRX066253	Bowel adenocarcinoma tissue	Primary tumor
	SRR222178	SRX066255	Bowel adenocarcinoma tissue	Primary tumor
	SRR5080874[16]	SRX2399057	Human colorectal cancer HT-29 cells	Tumor cell line
	SRR5080875[16]	SRX2399058	Human colorectal cancer HT-29 cells	Tumor cell line
	SRR5080876[16]	SRX2399060	Human colorectal cancer HT-29 cells	Tumor cell line
**Pancreatic Cancer PDX**	ERR1084829[2]	ERX1164419	Pancreatic Cancer	PDX tumor
	ERR1084830[2]	ERX1164420	Pancreatic Cancer	PDX tumor
	ERR2603556	ERX2620212	Pancreatic ductal adenocarcinoma	PDX tumor, CB17 SCID
	ERR2603557	ERX2620213	Pancreatic ductal adenocarcinoma	PDX tumor, CB17 SCID
	ERR2603558	ERX2620214	Pancreatic ductal adenocarcinoma	PDX tumor, nude mouse
	ERR2603559	ERX2620215	Pancreatic ductal adenocarcinoma	PDX tumor, nude mouse
	ERR2603560	ERX2620216	Pancreatic ductal adenocarcinoma	PDX tumor, nude mouse
	ERR2603561	ERX2620217	Pancreatic ductal adenocarcinoma	PDX tumor, nude mouse
	ERR2603562	ERX2620218	Pancreatic ductal adenocarcinoma	PDX tumor, nude mouse
	ERR2603563	ERX2620219	Pancreatic ductal adenocarcinoma	PDX tumor, nude mouse
	ERR2603564	ERX2620220	Pancreatic ductal adenocarcinoma	PDX tumor, nude mouse
	ERR2603565	ERX2620221	Pancreatic ductal adenocarcinoma	PDX tumor, nude mouse
	ERR2603566	ERX2620222	Pancreatic ductal adenocarcinoma	PDX tumor, nude mouse
	ERR2603567	ERX2620223	Pancreatic ductal adenocarcinoma	PDX tumor, nude mouse
	ERR2603568	ERX2620224	Pancreatic ductal adenocarcinoma	PDX tumor, nude mouse
	ERR2603569	ERX2620225	Pancreatic ductal adenocarcinoma	PDX tumor, nude mouse
**Pancreatic Cancer Control**	SRR3308883[17]	SRX1668055	Pancreatic adenocarcinoma cancer tissue	Primary tumor
	SRR3308884[17]	SRX1668056	Pancreatic adenocarcinoma cancer tissue	Primary tumor
	SRR3308885[17]	SRX1668057	Pancreatic adenocarcinoma cancer tissue	Primary tumor
	SRR3308886[17]	SRX1668058	Pancreatic adenocarcinoma cancer tissue	Primary tumor
	SRR3308887[17]	SRX1668059	Pancreatic adenocarcinoma cancer tissue	Primary tumor
	SRR3308888[17]	SRX1668060	Pancreatic adenocarcinoma cancer tissue	Primary tumor

	SRR3308889[17]	SRX1668061	Pancreatic adenocarcinoma cancer tissue	Primary tumor
	SRR3308891[17]	SRX1668063	Pancreatic adenocarcinoma cancer tissue	Primary tumor
	SRR3308892[17]	SRX1668064	Pancreatic adenocarcinoma cancer tissue	Primary tumor
	SRR3308893[17]	SRX1668065	Pancreatic adenocarcinoma cancer tissue	Primary tumor
	SRR3308894[17]	SRX1668066	Pancreatic adenocarcinoma cancer tissue	Primary tumor
	SRR3308895[17]	SRX1668067	Pancreatic adenocarcinoma cancer tissue	Primary tumor
	SRR3308896[17]	SRX1668068	Pancreatic adenocarcinoma cancer tissue	Primary tumor
	SRR3308897[17]	SRX1668069	Pancreatic adenocarcinoma cancer tissue	Primary tumor
	SRR3308898[17]	SRX1668070	Pancreatic adenocarcinoma cancer tissue	Primary tumor
	SRR3308899[17]	SRX1668071	Pancreatic adenocarcinoma cancer tissue	Primary tumor
	SRR3308900[17]	SRX1668072	Pancreatic adenocarcinoma cancer tissue	Primary tumor
	SRR3308901[17]	SRX1668073	Pancreatic adenocarcinoma cancer tissue	Primary tumor
	SRR3308902[17]	SRX1668074	Pancreatic adenocarcinoma cancer tissue	Primary tumor
	SRR3308903[17]	SRX1668075	Pancreatic adenocarcinoma cancer tissue	Primary tumor
	SRR3308904[17]	SRX1668076	Pancreatic adenocarcinoma cancer tissue	Primary tumor
	SRR3308905[17]	SRX1668077	Pancreatic adenocarcinoma cancer tissue	Primary tumor
	SRR3308906[17]	SRX1668078	Pancreatic adenocarcinoma cancer tissue	Primary tumor
	SRR3308907[17]	SRX1668079	Pancreatic adenocarcinoma cancer tissue	Primary tumor
	SRR3308908[17]	SRX1668080	Pancreatic adenocarcinoma cancer tissue	Primary tumor
	SRR3308909[17]	SRX1668081	Pancreatic adenocarcinoma cancer tissue	Primary tumor
	SRR3308910[17]	SRX1668082	Pancreatic adenocarcinoma cancer tissue	Primary tumor
	SRR3308911[17]	SRX1668083	Pancreatic adenocarcinoma cancer tissue	Primary tumor
	SRR3308912[17]	SRX1668084	Pancreatic adenocarcinoma cancer tissue	Primary tumor
	SRR3308913[17]	SRX1668085	Pancreatic adenocarcinoma cancer tissue	Primary tumor
	SRR3308914[17]	SRX1668086	Pancreatic adenocarcinoma cancer tissue	Primary tumor
	SRR3308915[17]	SRX1668087	Pancreatic adenocarcinoma cancer tissue	Primary tumor
	SRR3308916[17]	SRX1668088	Pancreatic adenocarcinoma cancer tissue	Primary tumor
	SRR3308917[17]	SRX1668089	Pancreatic adenocarcinoma cancer tissue	Primary tumor
	SRR3308918[17]	SRX1668090	Pancreatic adenocarcinoma cancer tissue	Primary tumor
	SRR3308919[17]	SRX1668091	Pancreatic adenocarcinoma cancer tissue	Primary tumor
	SRR3308920[17]	SRX1668092	Pancreatic adenocarcinoma cancer tissue	Primary tumor
	SRR3308921[17]	SRX1668093	Pancreatic adenocarcinoma cancer tissue	Primary tumor
	SRR3308922[17]	SRX1668094	Pancreatic adenocarcinoma cancer tissue	Primary tumor
	SRR3308923[17]	SRX1668095	Pancreatic adenocarcinoma cancer tissue	Primary tumor
	SRR3308924[17]	SRX1668096	Pancreatic adenocarcinoma cancer tissue	Primary tumor
	SRR3308925[17]	SRX1668097	Pancreatic adenocarcinoma cancer tissue	Primary tumor
	SRR3308926[17]	SRX1668098	Pancreatic adenocarcinoma cancer tissue	Primary tumor
	SRR3308927[17]	SRX1668099	Pancreatic adenocarcinoma cancer tissue	Primary tumor
	SRR3308928[17]	SRX1668100	Pancreatic adenocarcinoma cancer tissue	Primary tumor
	SRR3308929[17]	SRX1668101	Pancreatic adenocarcinoma cancer tissue	Primary tumor
	SRR3308930[17]	SRX1668102	Pancreatic adenocarcinoma cancer tissue	Primary tumor
	SRR3308931[17]	SRX1668103	Pancreatic adenocarcinoma cancer tissue	Primary tumor
	SRR3308932[17]	SRX1668104	Pancreatic adenocarcinoma cancer tissue	Primary tumor
**Wild Type Mice**	SRR5318208	SRX2617674		
	SRR5318209	SRX2617675		
	SRR7425025[18]	SRX4295851		
	SRR7425026[18]	SRX4295852		
	SRR7425027[18]	SRX4295853		
**NOD scid gamma Mice**	SRR570445[19]	SRX187137		
	SRR570446[19]	SRX187138		
	SRR570447[19]	SRX187139		
	SRR7421917	SRX4292742		
	SRR7421918	SRX4292743		
	SRR7421919	SRX4292744		
	SRR7421921	SRX4292746		
	SRR7421922	SRX4292747		
	SRR7421928	SRX4292753		
	SRR7421929	SRX4292754		
	SRR7421930	SRX4292755		
	SRR7421931	SRX4292756		
	SRR7421932	SRX4292757		
	SRR7421933	SRX4292759		
**EBV posititve**	SRR1178330[20]	SRX435680		

Supplementary 2c. Data used as positive control for our assembly method

PRJEB14936	Run Accession	Experiment Accession
**Zika virus**	ERR1549324	ERX1620094
	ERR1549325	ERX1620095

	ERR1549326	ERX1620096
	ERR1549327	ERX1620097
	ERR1549329	ERX1620099

Supplementary Table 3. The murine originated viruses and their hosts. The hosts of the viruses are validated from the literature and databases.

Virus	Host	References
Abelson murine leukemia virus	*Mus musculus*	[21]
AKR (endogenous) murine leukemia virus	*Mus musculus*	[21]
AKT8 retrovirus	*Mus musculus*	[22]
Curionopolis virus	Culicoides/*Mus musculus*	[23]
Endogenous mouse mammary tumor virus Mtv1	*Mus musculus*	[24]
Exogenous mouse mammary tumor virus	*Mus musculus*	[21]
Friend spleen focus-forming virus	*Mus musculus*	[21]
Harvey murine sarcoma virus	*Mus musculus*	[21]
HoMuLV murine leukemia virus	*Mus musculus*	[25]
Intracisternal A-type particle IAP	*Mus musculus*/Human	[26]
Lactate dehydrogenase-elevating virus	*Mus musculus*	[21]
MLV-related virus CFS	*Mus musculus*	[27]
Moloney murine leukemia virus	*Mus musculus*	[21,28]
Moloney murine sarcoma virus	*Mus musculus*	[21]
Mouse mammary tumor virus	*Mus musculus*	[21]
Murine AIDS virus-related provirus	*Mus musculus*	[29]
Murine leukemia virus	*Mus musculus*/Human	[30]
Murine leukemia virus SL3-2	*Mus musculus*/Human/Mink	[31]
Murine norovirus	*Mus musculus*	[21]
Myeloproliferative leukemia virus	*Mus musculus*	[32]
Myeloproliferative sarcoma virus	*Mus musculus*	[33]
Rauscher murine leukemia virus	*Mus musculus*/primates (for xenotropic)	[21]
Recombinant M-MuLV/RaLV retrovirus	*Mus musculus*	[34,35]
Xenotropic murine leukemia virus	*Mus musculus/Homo sapiens*	[21]
xenotropic murine leukemia virus-related virus XMRV	*Mus musculus*	[27,36]

Supplementary Table 4. The summary of the mouse and virus reads of all the PDX samples used in the original study to generate the data. The human reads, the calibrated and normalized mouse reads, virus reads and their proportion in the total sequenced reads along with the murine virus percentage in virome are listed. The proportion of reads that can be mapped to human are calculated via STAR [37], the mouse host somatic reads are further calculated by bowtie2 [38]. The Mouse mapped Total adjusted for reads mapped to human genome (MT-HG) are calibrated by the ×1.127 of the mouse mapped total reads as described in the Supplementary Table 5. The MT-HG and Virus Reads are normalized by their respective mouse genomes and virus genome size for comparison.

Column	I	II	III	IV	V	VI	VII	VIII	IX	X	XI	XII	XIII	XIV
Breast Cancer PDX	Experiment Accession	Total Reads	Reads Unmapped to Human	Unmapped Reads (%)	Mouse mapped Total	Mouse mapped Total (adjusted for mouse reads mapped to human genome) (MT-HG)	MT-HG normalized by mouse genome size (MT-HG/2.7G)	MT-HG in Total Reads (%)	Total Virus Reads	Total virus Reads normalized by virus genome size (Virus Reads/7934)	Total Murine Virus Reads	Murine Virus in virome (%)	Reads not mapped to any genome	% not mapped at all
	ERX2603577	7236594	818816	11.31%	312794	352627.192	1.30E-04	4.87%	14026	1.77E+00	12567	89.60%	491996	6.80%
	ERX2603578	8667598	983064	11.34%	375460	423273.482	1.56E-04	4.88%	15928	2.01E+00	14983	94.07%	591676	6.83%
	ERX2603579	9387360	1058266	11.27%	411244	463614.445	1.71E-04	4.94%	18346	2.31E+00	16551	90.22%	628676	6.70%
	ERX2603580	8092792	1495812	18.48%	721126	812958.804	2.99E-04	10.05%	11845	1.49E+00	9001	75.99%	762841	9.43%
	ERX2603581	9577416	1769462	18.48%	851506	959942.229	3.53E-04	10.02%	13546	1.71E+00	10620	78.40%	904410	9.44%
	ERX2603582	10409664	1909228	18.34%	928224	1046429.990	3.85E-04	10.05%	15158	1.91E+00	11961	78.91%	965846	9.28%
	ERX2603583	7534654	1865050	24.75%	1072758	1209369.876	4.45E-04	16.05%	69195	8.72E+00	64826	93.69%	723097	9.60%
	ERX2603584	8543928	2115490	24.76%	1219270	1374539.652	5.06E-04	16.09%	76957	9.70E+00	72417	94.10%	819263	9.59%
	ERX2603585	9188834	2267342	24.67%	1308050	1474625.466	5.43E-04	16.05%	81021	1.02E+01	75842	93.61%	878271	9.56%
	ERX2603586	8701162	1426576	16.40%	667170	752131.702	2.77E-04	8.64%	40469	5.10E+00	36821	90.99%	718937	8.26%
	ERX2603587	10170986	1670848	16.43%	783956	883789.979	3.25E-04	8.69%	46328	5.84E+00	42902	92.60%	840564	8.26%
	ERX2603588	10885966	1775332	16.31%	838434	945205.557	3.48E-04	8.68%	48649	6.13E+00	44679	91.84%	888249	8.16%
	ERX2603589	7331734	897794	12.25%	327766	369505.822	1.36E-04	5.04%	24817	3.13E+00	23248	93.68%	545211	7.44%
	ERX2603590	8531550	1039970	12.19%	379930	428312.720	1.58E-04	5.02%	29033	3.66E+00	27013	93.04%	631007	7.40%
	ERX2603591	9180772	1114426	12.14%	410490	462764.426	1.70E-04	5.04%	33141	4.18E+00	31067	93.74%	670795	7.31%
	ERX2603592	8406656	1295834	15.41%	589154	664180.644	2.44E-04	7.90%	84262	1.06E+01	81077	96.22%	622418	7.40%
	ERX2603593	9677312	1491276	15.41%	677464	763736.606	2.81E-04	7.89%	96084	1.21E+01	92563	96.34%	717728	7.42%
	ERX2603594	10450454	1593602	15.25%	729398	822284.214	3.03E-04	7.87%	101339	1.28E+01	97406	96.12%	762865	7.30%
	ERX2603595	9186176	4212692	45.86%	2597192	2927935.067	1.08E-03	31.87%	158787	2.00E+01	148093	93.27%	1456713	15.86%
	ERX2603596	10594600	4858464	45.86%	2990912	3371793.894	1.24E-03	31.83%	181039	2.28E+01	168878	93.28%	1686513	15.92%

ERX2603597	11364030	5200952	45.77%	3208442	3617025.558	1.33E-03	31.83%	195144	2.46E+01	182188	93.36%	1797366	15.82%
ERX2603598	9137432	1011204	11.07%	423308	477214.752	1.76E-04	5.22%	64853	8.17E+00	62573	96.48%	523043	5.72%
ERX2603599	10652626	1178150	11.06%	498546	562034.041	2.07E-04	5.28%	74689	9.41E+00	72030	96.44%	604915	5.68%
ERX2603600	11471538	1256410	10.95%	532268	600050.417	2.21E-04	5.23%	83319	1.05E+01	79178	95.03%	640823	5.59%
ERX2603601	7003098	1765868	25.22%	1016302	1145724.407	4.22E-04	16.36%	47121	5.94E+00	43002	91.26%	702445	10.03%
ERX2603602	8176598	2061536	25.21%	1181960	1332478.358	4.90E-04	16.30%	53240	6.71E+00	48755	91.58%	826336	10.11%
ERX2603603	8827046	2221068	25.16%	1279844	1442827.534	5.31E-04	16.35%	58913	7.43E+00	53650	91.07%	882311	10.00%
ERX2603604	8042878	1349866	16.78%	696564	785268.922	2.89E-04	9.76%	24505	3.09E+00	21677	88.46%	628797	7.82%
ERX2603605	9279262	1555402	16.76%	805372	907933.230	3.34E-04	9.78%	27588	3.48E+00	24938	90.39%	722442	7.79%
ERX2603606	10003552	1671542	16.71%	868016	978554.718	3.60E-04	9.78%	28924	3.65E+00	25742	89.00%	774602	7.74%
ERX2603607	6886348	2174946	31.58%	1282110	1445382.101	5.32E-04	20.99%	42537	5.36E+00	36497	85.80%	850299	12.35%
ERX2603608	7968902	2516498	31.58%	1479362	1667753.434	6.14E-04	20.93%	48171	6.07E+00	41802	86.78%	988965	12.41%
ERX2603609	8689812	2738066	31.51%	1611548	1816772.846	6.69E-04	20.91%	52806	6.66E+00	45676	86.50%	1073712	12.36%
ERX2603610	2932390	562618	19.19%	328936	370824.817	1.36E-04	12.65%	60171	7.58E+00	58807	97.73%	173511	5.92%
ERX2603611	2235272	284072	12.71%	147748	166563.177	6.13E-05	7.45%	23261	2.93E+00	22421	96.39%	113063	5.06%
ERX2603612	2513352	323206	12.86%	170244	191923.962	7.06E-05	7.64%	36823	4.64E+00	35726	97.02%	116139	4.62%
ERR2587337	2206200	227020	10.29%	131354	148081.460	5.45E-05	6.71%	38750	4.88E+00	38037	98.16%	56916	2.58%
ERX2603614	3929478	484856	12.34%	250166	282023.741	1.04E-04	7.18%	32323	4.07E+00	30903	95.61%	202367	5.15%
ERX2603615	2313618	181646	7.85%	86802	97855.923	3.60E-05	4.23%	2870	3.62E-01	2558	89.13%	91974	3.98%
ERX2603616	2212406	261026	11.80%	155464	175261.782	6.45E-05	7.92%	51035	6.43E+00	50003	97.98%	54527	2.46%
ERX2603617	2616620	230170	8.80%	120814	136199.229	5.01E-05	5.21%	19505	2.46E+00	18978	97.30%	89851	3.43%
ERX2603618	2018532	314438	15.58%	181856	205014.708	7.55E-05	10.16%	74263	9.36E+00	72756	97.97%	58319	2.89%
ERX2603619	2402890	348082	14.49%	196682	221728.746	8.16E-05	9.23%	115533	1.46E+01	113502	98.24%	35867	1.49%
ERX2603620	22673850	2795794	12.33%	1055256	1189639.059	4.38E-04	5.25%	184614	2.33E+01	178410	96.64%	1555924	6.86%
SRX3700451	37505594	1446714	3.86%	925812	1043710.831	3.84E-04	2.78%	56891	7.17E+00	48897	85.95%	464011	1.24%
SRX3700452	27529700	1955218	7.10%	1265068	1426169.863	5.25E-04	5.18%	69161	8.72E+00	59100	85.45%	620989	2.26%
SRX3700453	32558100	1207240	3.71%	760628	857491.242	3.16E-04	2.63%	50808	6.40E+00	42569	83.78%	395804	1.22%
SRX3700454	32607230	1018456	3.12%	615246	693595.367	2.55E-04	2.13%	51765	6.52E+00	38751	74.86%	351445	1.08%

SRX3700455	32380074	1021288	3.15%	624746	704305.158	2.59E-04	2.18%	48652	6.13E+00	35219	72.39%	347890	1.07%
SRR6727453	32888868	1164432	3.54%	694686	783151.765	2.88E-04	2.38%	56522	7.12E+00	41265	73.01%	413224	1.26%
SRX3700457	34239528	1114980	3.26%	673636	759421.124	2.80E-04	2.22%	51595	6.50E+00	38139	73.92%	389749	1.14%
SRX3700458	33275136	1029208	3.09%	620554	699579.322	2.57E-04	2.10%	49551	6.25E+00	36418	73.50%	359103	1.08%
SRX3700459	36013580	1209386	3.36%	739876	834096.549	3.07E-04	2.32%	51809	6.53E+00	37671	72.71%	417701	1.16%
SRX3700460	43865730	18281992	41.68%	14198128	16006208.573	5.89E-03	36.49%	82415	1.04E+01	19791	24.01%	4001449	9.12%
SRX3700461	31343796	2360082	7.53%	1585980	1787948.853	6.58E-04	5.70%	67768	8.54E+00	45911	67.75%	706334	2.25%
SRX3700462	33951484	2769500	8.16%	1858772	2095479.934	7.71E-04	6.17%	75803	9.55E+00	55284	72.93%	834925	2.46%
SRX3700463	27910946	2153182	7.71%	1412226	1592067.906	5.86E-04	5.70%	64259	8.10E+00	44764	69.66%	676697	2.42%
SRX3700464	32480222	3963776	12.20%	2680730	3022111.331	1.11E-03	9.30%	122043	1.54E+01	65272	53.48%	1161003	3.57%
SRX3700465	29488940	5178558	17.56%	3397526	3830188.725	1.41E-03	12.99%	113009	1.42E+01	78529	69.49%	1668023	5.66%
SRX3700466	27337348	4891474	17.89%	3156848	3558861.247	1.31E-03	13.02%	102239	1.29E+01	67170	65.70%	1632387	5.97%
SRX3700467	30854846	3708424	12.02%	2410452	2717414.399	1.00E-03	8.81%	107361	1.35E+01	61648	57.42%	1190611	3.86%
SRX3700468	31508866	4571656	14.51%	3073626	3465041.224	1.28E-03	11.00%	119533	1.51E+01	83881	70.17%	1378497	4.37%
SRX3700469	56453616	16498918	29.23%	10752102	12121343.547	4.46E-03	21.47%	643758	8.11E+01	559704	86.94%	5103058	9.04%
SRX3700470	36813714	26826772	72.87%	18037472	20334479.233	7.48E-03	55.24%	106701	1.34E+01	19388	18.17%	8682599	23.59%
SRX3700471	36172436	6425524	17.76%	3530164	3979717.698	1.46E-03	11.00%	118004	1.49E+01	83180	70.49%	2777356	7.68%
ERX1164353	101579514	5713622	5.62%	2929062	3302067.519	1.22E-03	3.25%	302511	3.81E+01	256319	84.73%	2482049	2.44%
ERX1164354	116305262	6359644	5.47%	3478702	3921702.197	1.44E-03	3.37%	362329	4.57E+01	307350	84.83%	2518613	2.17%
ERX1164355	115235174	7899814	6.86%	4463290	5031673.940	1.85E-03	4.37%	266976	3.36E+01	199522	74.73%	3169548	2.75%
ERX1164356	85372966	12730934	14.91%	8970292	10112626.447	3.72E-03	11.85%	1152385	1.45E+02	1086545	94.29%	2608257	3.06%
ERX1164357	99435122	5223460	5.25%	2977934	3357163.192	1.24E-03	3.38%	1713828	2.16E+02	1408653	82.19%	531698	0.53%
ERX1164358	111624706	5307206	4.75%	2697002	3040455.512	1.12E-03	2.72%	210384	2.65E+01	162786	77.38%	2399820	2.15%
ERX1164365	128870256	15841810	12.29%	10090326	11375292.751	4.19E-03	8.83%	448927	5.66E+01	322452	71.83%	5302557	4.11%
ERX1164388	112074810	7910036	7.06%	4469236	5038377.142	1.85E-03	4.50%	77074	9.71E+00	11148	14.46%	3363726	3.00%
ERX1164389	94866644	9875008	10.41%	5357756	6040046.971	2.22E-03	6.37%	3570111	4.50E+02	3253951	91.14%	947141	1.00%
ERX1164390	95786398	8812530	9.20%	5553116	6260285.365	2.30E-03	6.54%	3317084	4.18E+02	3082090	92.92%	-57670	-0.06%
ERX1164391	104135236	6009012	5.77%	3291200	3710322.491	1.37E-03	3.56%	124571	1.57E+01	78423	62.95%	2593241	2.49%

	ERX1164392	115393036	24030632	20.83%	16370708	18455458.827	6.79E-03	15.99%	163288	2.06E+01	37525	22.98%	7496636	6.50%
	ERX1164393	108812848	9568744	8.79%	5882910	6632077.445	2.44E-03	6.09%	221143	2.79E+01	153358	69.35%	3464691	3.18%
	ERX1164394	115758420	9940022	8.59%	6028484	6796189.771	2.50E-03	5.87%	250800	3.16E+01	183124	73.02%	3660738	3.16%
	ERX1164395	127048038	14618060	11.51%	9333752	10522371.772	3.87E-03	8.28%	1158354	1.46E+02	946459	81.71%	4125954	3.25%
	ERX1164396	116648416	5820248	4.99%	2709130	3054127.969	1.12E-03	2.62%	39232	4.94E+00	9733	24.81%	3071886	2.63%
	ERX1164397	115298828	5614782	4.87%	2764050	3116041.834	1.15E-03	2.70%	287089	3.62E+01	223170	77.74%	2563643	2.22%
	ERX1164398	110156880	27895172	25.32%	19984868	22529869.114	8.29E-03	20.45%	3842680	4.84E+02	3670974	95.53%	4067624	3.69%
	ERX1164399	105802220	8390396	7.93%	4846166	5463307.823	2.01E-03	5.16%	293451	3.70E+01	249653	85.07%	3250779	3.07%
	ERX1164400	85429324	8284440	9.70%	5816210	6556883.440	2.41E-03	7.68%	1347766	1.70E+02	1070883	79.46%	1120464	1.31%
	ERX1164401	106142076	9553258	9.00%	5563354	6271827.137	2.31E-03	5.91%	646802	8.15E+01	561862	86.87%	3343102	3.15%

Glioblastoma PDX	Experiment Accession	Total Reads	Reads Unmapped to Human	Unmapped Reads (%)	Mouse mapped Total	Mouse mapped Total (adjusted for mouse reads mapped to human genome) （MT-HG）	MT-HG normalized by mouse genome size (MT-HG/2.7G)	MT-HG in Total Reads (%)	Total Virus Reads	Total virus Reads normalized by virus genome size (Virus Reads/7934)	Total Murine Virus Reads	Murine Virus in virome (%)	Reads not mapped to any genome	% not mapped at all
	SRX2248852	66935032	2713472	4.05%	527756	594963.830	2.19E-04	0.89%	880574	1.11E+02	829108	94.16%	1305142	1.95%
	SRX2248853	60158040	3176852	5.28%	1047348	1180724.004	4.35E-04	1.96%	1453339	1.83E+02	1379052	94.89%	676165	1.12%
	SRX2248854	63601076	2678300	4.21%	810852	914111.088	3.36E-04	1.44%	1164012	1.47E+02	1096620	94.21%	703436	1.11%
	SRX2248855	68561012	1817500	2.65%	203068	228927.980	8.43E-05	0.33%	266549	3.36E+01	246206	92.37%	1347883	1.97%
	SRX2248856	61890646	1635214	2.64%	175820	198210.045	7.30E-05	0.32%	226244	2.85E+01	206044	91.07%	1233150	1.99%

Lung PDX	Experiment Accession	Total Reads	Reads Unmapped to Human	Unmapped Reads (%)	Mouse mapped Total	Mouse mapped Total (adjusted for mouse reads mapped to human genome) （MT-HG）	MT-HG normalized by mouse genome size (MT-HG/2.7G)	MT-HG in Total Reads (%)	Total Virus Reads	Total virus Reads normalized by virus genome size (Virus Reads/7934)	Total Murine Virus Reads	Murine Virus in virome (%)	Reads not mapped to any genome	% not mapped at all
	ERX1164366	124715422	4359998	3.50%	2716056	3061935.970	1.13E-03	2.46%	335980	4.23E+01	300842	89.54%	1307962	1.05%
	ERX1164367	124514854	6609340	5.31%	4128204	4653915.943	1.71E-03	3.74%	308100	3.88E+01	236024	76.61%	2173036	1.75%
	ERX1164368	102038424	4467156	4.38%	2537454	2860589.647	1.05E-03	2.80%	62930	7.93E+00	8918	14.17%	1866772	1.83%
	ERX1164369	120051702	16802738	14.00%	14462484	16304229.359	6.00E-03	13.58%	26807682	3.38E+03	25807181	96.27%	-24467428	-20.38%
	ERX1164370	128078496	4573414	3.57%	2972698	3351260.406	1.23E-03	2.62%	4201736	5.30E+02	3839432	91.38%	-2601020	-2.03%
	ERX1164374	131878658	17366848	13.17%	12257412	13818349.365	5.09E-03	10.48%	516371	6.51E+01	368906	71.44%	4593065	3.48%
	ERX1164375	107487630	5342360	4.97%	3502170	3948158.763	1.45E-03	3.67%	306281	3.86E+01	252286	82.37%	1533909	1.43%
	ERX1164376	114290342	10183044	8.91%	6232784	7026506.642	2.59E-03	6.15%	242426	3.06E+01	147963	61.03%	3707834	3.24%
	ERX1164377	126831612	11435466	9.02%	8017648	9038666.657	3.33E-03	7.13%	927400	1.17E+02	831500	89.66%	2490418	1.96%

ERX1164378	114857908	7042308	6.13%	4599650	5185398.896	1.91E-03	4.51%	1331346	1.68E+02	784151	58.90%	1111312	0.97%
ERX1164379	129914504	35268404	27.15%	26247630	29590171.347	1.09E-02	22.78%	681168	8.59E+01	460435	67.59%	8339606	6.42%
ERX1164380	114813252	7037116	6.13%	4428962	4992974.393	1.84E-03	4.35%	278599	3.51E+01	214754	77.08%	2329555	2.03%
ERX1164381	103696436	7745862	7.47%	4912856	5538490.555	2.04E-03	5.34%	365430	4.61E+01	285763	78.20%	2467576	2.38%
ERX1164382	126716746	7481196	5.90%	5085328	5732926.244	2.11E-03	4.52%	514632	6.49E+01	454430	88.30%	1881236	1.48%
ERX1164383	106461898	16283790	15.30%	11509454	12975141.602	4.78E-03	12.19%	1085872	1.37E+02	943476	86.89%	3688464	3.46%
ERX1164384	120999542	11285162	9.33%	7362258	8299815.096	3.05E-03	6.86%	353480	4.46E+01	248975	70.44%	3569424	2.95%
ERX1164385	113228010	11509608	10.16%	7867208	8869068.664	3.26E-03	7.83%	561220	7.07E+01	439595	78.33%	3081180	2.72%
ERX1164386	114700660	9678664	8.44%	6602514	7443320.428	2.74E-03	6.49%	473147	5.96E+01	386746	81.74%	2603003	2.27%
ERX1164387	111346280	17110510	15.37%	12264352	13826173.149	5.09E-03	12.42%	1222035	1.54E+02	1067813	87.38%	3624123	3.25%
ERX1164405	118721064	12624092	10.63%	8230252	9278345.013	3.41E-03	7.82%	148284	1.87E+01	47840	32.26%	4245556	3.58%
ERX1164406	105773672	39276560	37.13%	27916986	31472113.833	1.16E-02	29.75%	2260690	2.85E+02	43611	1.93%	9098884	8.60%
ERX1164407	110560642	14205466	12.85%	9454040	10657978.016	3.92E-03	9.64%	294361	3.71E+01	155940	52.98%	4457065	4.03%
ERX1164408	112658272	11355808	10.08%	8024548	9046445.348	3.33E-03	8.03%	330802	4.17E+01	237619	71.83%	3000458	2.66%
ERX1164409	131325820	19428524	14.79%	13652716	15391340.315	5.66E-03	11.72%	457862	5.77E+01	345504	75.46%	5317946	4.05%
ERX1164410	123309538	40261594	32.65%	29856014	33658070.080	1.24E-02	27.30%	742896	9.36E+01	43343	5.83%	9662684	7.84%
ERX1164411	97960220	18786906	19.18%	13240408	14926526.373	5.49E-03	15.24%	411062	5.18E+01	307372	74.78%	5135436	5.24%
ERX1164412	108008052	17076992	15.81%	12573862	14175098.135	5.22E-03	13.12%	809806	1.02E+02	719353	88.83%	3693324	3.42%
ERX1164421	101440260	4206322	4.15%	2537008	2860086.851	1.05E-03	2.82%	111226	1.40E+01	78835	70.88%	1558088	1.54%
ERX1164422	115361300	24764158	21.47%	17982062	20272012.968	7.46E-03	17.57%	1270319	1.60E+02	1087921	85.64%	5511777	4.78%
ERX1164423	108898274	6592860	6.05%	2184340	2462507.848	9.06E-04	2.26%	216909	2.73E+01	182939	84.34%	4191611	3.85%
ERX1164424	122738356	7413428	6.04%	4120508	4645239.885	1.71E-03	3.78%	41825	5.27E+00	6346	15.17%	3251095	2.65%
ERX1164425	110771756	10047522	9.07%	6376118	7188093.712	2.65E-03	6.49%	139619	1.76E+01	20813	14.91%	3531785	3.19%
ERX1164426	100214040	12729444	12.70%	8289236	9344840.413	3.44E-03	9.32%	346587	4.37E+01	176167	50.83%	4093621	4.08%
ERX1164427	112866084	30332014	26.87%	20977784	23649229.398	8.70E-03	20.95%	1573739	1.98E+02	1356974	86.23%	7780491	6.89%
ERX1164428	83371946	4068176	4.88%	2557918	2883659.664	1.06E-03	3.46%	280857	3.54E+01	253346	90.20%	1229401	1.47%
ERX1164429	108850402	18584792	17.07%	12968316	14619784.434	5.38E-03	13.43%	1176386	1.48E+02	982397	83.51%	4440090	4.08%
ERX1164433	131552118	15254878	11.60%	10197096	11495659.527	4.23E-03	8.74%	261513	3.30E+01	121044	46.29%	4796269	3.65%

	SRX1098160	24605632	5283020	21.47%	2814964	3173439.548	1.17E-03	12.90%	204058	2.57E+01	116112	56.90%	2263998	9.20%

Ovarian Cancer PDX	Experiment Accession	Total Reads	Reads Unmapped to Human	Unmapped Reads (%)	Mouse mapped Total	Mouse mapped Total (adjusted for mouse reads mapped to human genome) （MT-HG）	MT-HG normalized by mouse genome size (MT-HG/2.7G)	MT-HG in Total Reads (%)	Total Virus Reads	Total virus Reads normalized by virus genome size (Virus Reads/7934)	Total Murine Virus Reads	Murine Virus in virome (%)	Reads not mapped to any genome	% not mapped at all
	ERX1164413	104388834	18027408	17.27%	13464436	15179083.533	5.59E-03	14.54%	4967067	6.26E+02	4064628	81.83%	-404095	-0.39%
	ERX1164414	97078034	3487822	3.59%	1578466	1779477.972	6.55E-04	1.83%	74120	9.34E+00	50074	67.56%	1835236	1.89%
	ERX1164415	105329632	68872064	65.39%	53339712	60132332.620	2.21E-02	57.09%	6001274	7.56E+02	5564999	92.73%	9531078	9.05%
	ERX1164416	116346896	9278094	7.97%	5756512	6489583.114	2.39E-03	5.58%	413048	5.21E+01	345220	83.58%	3108534	2.67%
	ERX1164430	82936248	5269420	6.35%	3477016	3919801.491	1.44E-03	4.73%	462914	5.83E+01	418633	90.43%	1329490	1.60%
	ERX1164431	118316926	13237628	11.19%	8516230	9600741.283	3.53E-03	8.11%	229032	2.89E+01	134074	58.54%	4492366	3.80%
	ERX1164432	84876878	6395696	7.54%	4144638	4672442.753	1.72E-03	5.50%	258847	3.26E+01	227747	87.99%	1992211	2.35%

Bladder Cancer PDX	Experiment Accession	Total Reads	Reads Unmapped to Human	Unmapped Reads (%)	Mouse mapped Total	Mouse mapped Total (adjusted for mouse reads mapped to human genome) （MT-HG）	MT-HG normalized by mouse genome size (MT-HG/2.7G)	MT-HG in Total Reads (%)	Total Virus Reads	Total virus Reads normalized by virus genome size (Virus Reads/7934)	Total Murine Virus Reads	Murine Virus in virome (%)	Reads not mapped to any genome	% not mapped at all
	SRX3003255	52528724	5806708	11.05%	3267628	3683748.682	1.36E-03	7.01%	404490	5.10E+01	356339	88.10%	2134590	4.06%
	SRX3003256	53513770	8438358	15.77%	5034032	5675097.883	2.09E-03	10.60%	487213	6.14E+01	410797	84.32%	2917113	5.45%
	SRX3003257	54927930	7180542	13.07%	3725700	4200154.505	1.55E-03	7.65%	320101	4.03E+01	208965	65.28%	3134741	5.71%
	SRX3003258	47839218	6804950	14.22%	3766112	4245712.828	1.56E-03	8.87%	304168	3.83E+01	246742	81.12%	2734670	5.72%
	SRX3003259	47655968	7823416	16.42%	4528736	5105454.253	1.88E-03	10.71%	371491	4.68E+01	247602	66.65%	2923189	6.13%
	SRX3003260	59280530	10099048	17.04%	5872210	6620014.839	2.44E-03	11.17%	1027309	1.29E+02	165126	16.07%	3199529	5.40%
	SRX3003261	57147356	9947292	17.41%	5845270	6589644.127	2.43E-03	11.53%	538985	6.79E+01	469390	87.09%	3563037	6.23%
	SRX3003262	47706782	5671846	11.89%	2958734	3335518.141	1.23E-03	6.99%	297560	3.75E+01	207776	69.83%	2415552	5.06%
	SRX3003263	49979236	14708622	29.43%	8937196	10075315.79	3.71E-03	20.16%	653855	8.24E+01	548348	83.86%	5117571	10.24%
	SRX4283514	30638905	2508285	8.19%	2118388	2388157.098	8.79E-04	7.79%	749666	9.45E+01	595546	79.44%	-359769	-1.17%
	SRX4283515	23837568	1601671	6.72%	1351147	1523210.714	5.61E-04	6.39%	414315	5.22E+01	288673	69.67%	-163791	-0.69%
	SRX4283516	32376254	2303967	7.12%	1946873	2194800.279	8.08E-04	6.78%	582372	7.34E+01	420551	72.21%	-225278	-0.70%
	SRX4283517	32748959	702526	2.15%	423041	476913.7509	1.76E-04	1.46%	161812	2.04E+01	4011	2.48%	117673	0.36%
	SRX4283518	25888796	452236	1.75%	259209	292218.3346	1.08E-04	1.13%	109114	1.38E+01	5251	4.81%	83913	0.32%
	SRX4283519	35950185	616556	1.72%	332440	374775.0392	1.38E-04	1.04%	149243	1.88E+01	6695	4.49%	134873	0.38%
	SRX4283520	37265842	3043459	8.17%	2476713	2792113.499	1.03E-03	7.49%	315678	3.98E+01	145942	46.23%	251068	0.67%

	SRX4283521	29825519	2402256	8.05%	1948022	2196095.601	8.08E-04	7.36%	260360	3.28E+01	119254	45.80%	193874	0.65%
	SRX4283522	39792870	3291966	8.27%	2684010	3025809.027	1.11E-03	7.60%	346720	4.37E+01	166384	47.99%	261236	0.66%
	SRX4283523	13081572	816423	6.24%	638966	720336.0237	2.65E-04	5.51%	600623	7.57E+01	417237	69.47%	-423166	-3.23%
	SRX4283524	22697913	1255652	5.53%	980748	1105642.733	4.07E-04	4.87%	1266020	1.60E+02	884986	69.90%	-991116	-4.37%
	SRX4283525	23741982	1672316	7.04%	1336880	1507126.863	5.55E-04	6.35%	1265672	1.60E+02	893206	70.57%	-930236	-3.92%
	SRX4283526	28488281	669366	2.35%	424416	478463.8522	1.76E-04	1.68%	129243	1.63E+01	13998	10.83%	115707	0.41%
	SRX4283527	29512496	642661	2.18%	396138	446584.7506	1.64E-04	1.51%	124965	1.58E+01	12466	9.98%	121558	0.41%
	SRX4283528	25796017	583796	2.26%	372704	420166.5149	1.55E-04	1.63%	115691	1.46E+01	11723	10.13%	95401	0.37%
**Colorectal Cancer PDX**	**Experiment Accession**	**Total Reads**	**Reads Unmapped to Human**	**Unmapped Reads (%)**	**Mouse mapped Total**	**Mouse mapped Total (adjusted for mouse reads mapped to human genome) （MT-HG）**	**MT-HG normalized by mouse genome size (MT-HG/2.7G)**	**MT-HG in Total Reads (%)**	**Total Virus Reads**	**Total virus Reads normalized by virus genome size (Virus Reads/7934)**	**Total Murine Virus Reads**	**Murine Virus in virome (%)**	**Reads not mapped to any genome**	**% not mapped at all**
	ERX1164359	83804634	9001802	10.74%	6527876	7359177.547	2.71E-03	8.78%	473567	5.97E+01	424303	89.60%	2000359	2.39%
	ERX1164360	83662226	19369198	23.15%	14342594	16169071.799	5.95E-03	19.33%	3847817	4.85E+02	3646952	94.78%	1178787	1.41%
	ERX1164361	85262398	6889430	8.08%	4443244	5009075.154	1.84E-03	5.87%	84891	1.07E+01	50329	59.29%	2361295	2.77%
	ERX1164362	85300946	6126452	7.18%	4138146	4665124.021	1.72E-03	5.47%	98109	1.24E+01	66761	68.05%	1890197	2.22%
	ERX1164363	85062400	4938140	5.81%	3199566	3607019.231	1.33E-03	4.24%	315260	3.97E+01	287394	91.16%	1423314	1.67%
	ERX1164364	84447816	9484676	11.23%	6589406	7428543.172	2.73E-03	8.80%	321814	4.06E+01	281070	87.34%	2573456	3.05%
	ERX1164402	85676212	4158378	4.85%	2811890	3169974.086	1.17E-03	3.70%	1451340	1.83E+02	1181242	81.39%	-104852	-0.12%
	ERX1164403	83620526	12906992	15.44%	8952968	10093096.298	3.71E-03	12.07%	286809	3.61E+01	241457	84.19%	3667215	4.39%
**Pancreatic Cancer PDX**	**Experiment Accession**	**Total Reads**	**Reads Unmapped to Human**	**Unmapped Reads (%)**	**Mouse mapped Total**	**Mouse mapped Total (adjusted for mouse reads mapped to human genome) （MT-HG）**	**MT-HG normalized by mouse genome size (MT-HG/2.7G)**	**MT-HG in Total Reads (%)**	**Total Virus Reads**	**Total virus Reads normalized by virus genome size (Virus Reads/7934)**	**Total Murine Virus Reads**	**Murine Virus in virome (%)**	**Reads not mapped to any genome**	**% not mapped at all**
	ERX1164419	85774612	12952518	15.10%	9246178	10423645.538	3.84E-03	12.15%	55254	6.96E+00	12635	22.87%	3651086	4.26%
	ERX1164420	84899278	6582606	7.75%	4696572	5294663.565	1.95E-03	6.24%	111908	1.41E+01	78564	70.20%	1774126	2.09%
	ERX2620212	18801263	3660954	19.47%	3259212	3674260.935	1.35E-03	19.54%	243684	3.07E+01	200705	82.36%	158058	0.84%
	ERX2620213	20532076	4183206	20.37%	3754011	4232070.809	1.56E-03	20.61%	310436	3.91E+01	240675	77.53%	118759	0.58%
	ERX2620214	28854638	8919688	30.91%	7857747	8858402.840	3.26E-03	30.70%	521200	6.57E+01	430965	82.69%	540741	1.87%
	ERX2620215	24839294	5576443	22.45%	5038641	5680293.822	2.09E-03	22.87%	454241	5.73E+01	384821	84.72%	83561	0.34%
	ERX2620216	27689904	8497605	30.69%	7657728	8632912.139	3.18E-03	31.18%	1047175	1.32E+02	950694	90.79%	-207298	-0.75%
	ERX2620217	18380543	5093553	27.71%	4499852	5072891.980	1.87E-03	27.60%	324987	4.10E+01	268050	82.48%	268714	1.46%

	ERX2620218	19834300	5237799	26.41%	4647688	5239554.363	1.93E-03	26.42%	379320	4.78E+01	324754	85.61%	210791	1.06%
	ERX2620219	22270601	2964709	13.31%	2651964	2989682.084	1.10E-03	13.42%	198168	2.50E+01	153682	77.55%	114577	0.51%
	ERX2620220	19103986	4176348	21.86%	3698931	4169976.569	1.53E-03	21.83%	386692	4.87E+01	329763	85.28%	90725	0.47%
	ERX2620221	16949918	5637104	33.26%	5164121	5821753.249	2.14E-03	34.35%	763821	9.63E+01	700821	91.75%	-290838	-1.72%
	ERX2620222	20629653	7523617	36.47%	6750531	7610186.861	2.80E-03	36.89%	476891	6.01E+01	399361	83.74%	296195	1.44%
	ERX2620223	25266317	6677288	26.43%	5963923	6723407.160	2.47E-03	26.61%	274620	3.46E+01	176171	64.15%	438745	1.74%
	ERX2620224	21321535	5992630	28.11%	5334140	6013423.558	2.21E-03	28.20%	591967	7.46E+01	518944	87.66%	66523	0.31%
	ERX2620225	19660817	4313617	21.94%	3804659	4289168.650	1.58E-03	21.82%	265559	3.35E+01	214851	80.91%	243399	1.24%

Derived Cell Cultures	Experiment Accession	Total Reads	Reads Unmapped to Human	Unmapped Reads (%)	Mouse mapped Total	Mouse mapped Total (adjusted for mouse reads mapped to human genome) （MT-HG）	MT-HG normalized by mouse genome size (MT-HG/2.7G)	MT-HG in Total Reads (%)	Total Virus Reads	Total virus Reads normalized by virus genome size (Virus Reads/7934)	Total Murine Virus Reads	Murine Virus in virome (%)	Reads not mapped to any genome	% not mapped at all
	SRR5114302	68477052	919236	1.34%	2356	2655.212	9.77E-07	0.0039%	5937	7.48E-01	1195	20.13%	910943	1.33%
	SRR5114303	57801610	1088348	1.88%	1634	1841.518	6.78E-07	0.0032%	5237	6.60E-01	968	18.48%	1081477	1.87%
	SRR5114304	49270294	736096	1.49%	1510	1701.77	6.26E-07	0.0035%	4521	5.70E-01	1647	36.43%	730065	1.48%
	SRR5114305	58455008	1113162	1.90%	1514	1706.278	6.28E-07	0.0029%	7575	9.55E-01	641	8.46%	1104073	1.89%
	SRR5114306	57487988	1057484	1.84%	1520	1713.04	6.30E-07	0.0030%	2599	3.28E-01	653	25.13%	1053365	1.83%
	SRR5114307	63836716	809190	1.27%	2068	2330.636	8.58E-07	0.0037%	12438	1.57E+00	860	6.91%	794684	1.24%
	SRR5114308	60288730	910028	1.51%	1526	1719.802	6.33E-07	0.0029%	2146	2.70E-01	411	19.15%	906356	1.50%
	SRR5114309	59163106	783294	1.32%	1732	1951.964	7.18E-07	0.0033%	6858	8.64E-01	919	13.40%	774704	1.31%
	SRR5114310	58896706	829506	1.41%	1758	1981.266	7.29E-07	0.0034%	7445	9.38E-01	512	6.88%	820303	1.39%
	SRR5114311	46563648	826564	1.78%	1432	1613.864	5.94E-07	0.0035%	4907	6.18E-01	406	8.27%	820225	1.76%
	SRR5114312	49180504	890560	1.81%	1376	1550.752	5.71E-07	0.0032%	7694	9.70E-01	589	7.66%	881490	1.79%
	SRR5114313	55247934	1114524	2.02%	1434	1616.118	5.95E-07	0.0029%	4310	5.43E-01	696	16.15%	1108780	2.01%
	SRR5114316	57273954	1090024	1.90%	1326	1494.402	5.50E-07	0.0026%	5607	7.07E-01	735	13.11%	1083091	1.89%
	SRR5114317	53098256	1236738	2.33%	1806	2035.362	7.49E-07	0.0038%	6412	8.08E-01	1040	16.22%	1228520	2.31%
	SRR5114318	55058792	1008698	1.83%	1120	1262.24	4.65E-07	0.0023%	2278	2.87E-01	529	23.22%	1005300	1.83%
	SRR5114314	61864856	1207002	1.95%	1738	1958.726	7.21E-07	0.0032%	5591	7.05E-01	352	6.30%	1199673	1.94%
	SRR5114315	60250254	1040486	1.73%	1354	1525.958	5.62E-07	0.0025%	5901	7.44E-01	585	9.91%	1033231	1.71%
	SRR5114320	65794874	746632	1.13%	3226	3635.702	1.34E-06	0.0055%	733949	9.25E+01	814	0.11%	9457	0.01%

GBM Primary Tumor	Experiment Accession	Total Reads	Reads Unmapped to Human	Unmapped Reads (%)	Mouse mapped Total	Mouse mapped Total (adjusted for mouse reads mapped to human genome) （MT-HG）	MT-HG normalized by mouse genome size (MT-HG/2.7G)	MT-HG in Total Reads (%)	Total Virus Reads	Total virus Reads normalized by virus genome size (Virus Reads/7934)	Total Murine Virus Reads	Murine Virus in virome (%)	Reads not mapped to any genome	% not mapped at all
	SRR4343486	71452912	1744868	2.44%	49262	55518.274	2.04E-05	0.0777%	383497	4.83E+01	680	0.18%	1312109	1.84%
	SRR5289362	61561022	748278	1.22%	12822	14450.394	5.32E-06	0.0235%	8112	1.02E+00	537	6.62%	727344	1.18%
	SRR6371761	145816422	2346114	1.61%	28088	31655.176	1.17E-05	0.0217%	185245	2.33E+01	8940	4.83%	2132781	1.46%
	SRR6371762	122313130	1886818	1.54%	26042	29349.334	1.08E-05	0.0240%	166484	2.10E+01	7524	4.52%	1694292	1.39%
	SRR6371763	175993692	2331280	1.32%	31342	35322.434	1.30E-05	0.0201%	166334	2.10E+01	6267	3.77%	2133604	1.21%

Supplementary Table 5. The calibration ratio of the mouse specific reads that mapped to human and mouse genome. The RNA-seq reads of NSG mice were first mapped to human genome. The reads that cannot be mapped to human genome are further mapped to mouse genome. The average ratio of (mouse mapped reads + human mapped reads) / mouse mapped reads are calculated as calibration ratio of 1.127 to infer the exact mouse reads as used in Supplementary Table 4.

Accession	Reads mapped to Human	Reads mapped to Mouse	Average (mouse reads + human reads) -- Average (mouse reads)	Reads mapped to Mouse	Reads mapped to Human	Average (mouse reads + human reads) -- Average (mouse reads)
SRR570445	463361	1962271	1.127	2688221	25724	1.034
SRR570446	273066	1131192		1491925	51406	
SRR570447	431145	1819945		2385046	72858	
SRR7421917	3410715	13872603		24900408	2241847	
SRR7421918	1741955	19986700		21953282	925439	
SRR7421919	683612	7668489		10570675	1276032	
SRR7421920	3475374	14711593		21492364	1715691	
SRR7421921	1971259	10608347		17520752	1071690	
SRR7421922	1506396	14984662		19415508	1207842	
SRR7421928	2838001	25883787		30531887	469328	
SRR7421929	1122245	10527278		11514438	962020	
SRR7421930	771838	7360775		7786201	658957	
SRR7421931	3145515	20407418		26838105	359684	
SRR7421932	817235	8582783		10918240	810045	
SRR7421933	554257	7672040		9440787	614633	

Supplementary Table 6. The number of raw reads mapped to the housekeeping gene actin beta (*ACTB*) in both single cell RNA-Seq and conventional RNA-Seq.

Single Cell Sequencing		Conventional Sequencing	
Accession	# of Reads	Accession	# of Reads
SRR2049480	57434	ERR1084776	401259
SRR2049481	40380	ERR1084777	469930
SRR2049482	63837	ERR1084778	90517
SRR2049483	43170	ERR1084779	147614
SRR2049484	59955	ERR1084780	94552
SRR2049485	130262	ERR1084784	95975
SRR2049486	102492	ERR1084785	71821
SRR2049487	13932	ERR1084786	160371
SRR2049488	14464	ERR1084787	177593
SRR2049489	11309	ERR1084788	119490
SRR2049490	18555	ERR1084789	387606
SRR2049491	5943	ERR1084790	205189
SRR2049492	12948	ERR1084791	243054
SRR2049493	14582	ERR1084792	210829
SRR2049494	20545	ERR1084793	160645
SRR2049495	13514	ERR1084794	175499
SRR2049496	13202	ERR1084795	142655
SRR2049497	14837	ERR1084796	148185
SRR2049498	5187	ERR1084797	146864
SRR2049499	3829	ERR1084815	259775
SRR2049500	15228	ERR1084816	290465
SRR2049501	16872	ERR1084817	155805
SRR2049502	9740	ERR1084818	177862
SRR2049503	15737	ERR1084819	246289
SRR2049504	15976	ERR1084820	376655
SRR2049505	6664	ERR1084821	110750
SRR2049506	20967	ERR1084822	146337
SRR2049507	15852	ERR1084831	55739
SRR2049508	12866	ERR1084832	165557
SRR2049509	7692	ERR1084833	110013
SRR2049510	7600	ERR1084834	191519
SRR2049511	11747	ERR1084835	396412
SRR2049512	7120	ERR1084836	74248
SRR2049513	19281	ERR1084837	159991
SRR2049514	12566	ERR1084838	51463
SRR2049515	6186	ERR1084839	255774

SRR2049516	22981	ERR1084843	123688
SRR2049517	4771		
SRR2049518	15802		
SRR2049519	8920		
SRR2049520	15396		
SRR2049521	12363		
SRR2049522	11841		
SRR2049523	11397		
SRR2049524	10335		
SRR2049525	10551		
SRR2049526	14928		
SRR2049527	13999		
SRR2049528	12482		
SRR2049529	9674		
SRR2049530	10546		
SRR2049531	13729		
SRR2049532	16720		
SRR2049533	19672		
SRR2049534	7660		
SRR2049535	17394		
SRR2049390	17745		
SRR2049392	22114		
SRR2049393	16782		
SRR2049394	53295		
SRR2049395	58700		
SRR2049396	7361		
SRR2049397	16285		
SRR2049398	7924		
SRR2049399	22884		
SRR2049400	15485		
SRR2049401	9868		
SRR2049402	33421		
SRR2049403	1721		
SRR2049404	12426		
SRR2049405	6645		
SRR2049406	10268		
SRR2049407	23138		
SRR2049408	23190		
SRR2049409	11392		
SRR2049410	34089		
SRR2049411	24535		
SRR2049412	28004		
SRR2049413	16944		
SRR2049414	34617		

SRR2049415	10494
SRR2049416	8985
SRR2049417	12981
SRR2049418	1370
SRR2049419	13034
SRR2049420	17926
SRR2049421	12901
SRR2049422	19297
SRR2049423	2797
SRR2049424	7335
SRR2049425	3853
SRR2049426	8415
SRR2049427	6893
SRR2049428	13329
SRR2049429	26781
SRR2049536	77985
SRR2049539	104618
SRR2103637	40186
SRR2103638	48274
SRR2049430	65545
SRR2049431	33852
SRR2049432	56986
SRR2049433	59631
SRR2049434	42678
SRR2049435	42609
SRR2049436	77522
SRR2049437	17330
SRR2049438	18085
SRR2049439	13297
SRR2049440	7722
SRR2049441	17373
SRR2049442	5340
SRR2049443	12892
SRR2049444	7794
SRR2049445	4118
SRR2049446	11851
SRR2049447	10065
SRR2049448	6429
SRR2049449	5430
SRR2049450	7707
SRR2049451	4050
SRR2049452	8471
SRR2049453	1530
SRR2049454	14654

SRR2049455	4009	
SRR2049456	2282	
SRR2049457	11243	
SRR2049458	13294	
SRR2049459	4666	
SRR2049460	9576	
SRR2049461	9087	
SRR2049462	11516	
SRR2049463	10735	
SRR2049464	34656	
SRR2049465	19459	
SRR2049466	1467	
SRR2049467	8452	
SRR2049468	8426	
SRR2049469	8732	
SRR2049470	9878	
SRR2049471	27178	
SRR2049472	9534	
SRR2049473	10359	
SRR2049474	6189	
SRR2049475	12250	
SRR2049476	6437	
SRR2049477	11388	
SRR2049478	6233	
SRR2049479	10076	

Supplementary Table 7. The description of the PDX samples used to detect murine leukemia virus by PCR. The PDX samples, Pathology Diagnosis; DNA extraction concentration (ng/uL); A260; A280; 260/280; 260/230 are listed.

PDX samples	Pathology Diagnosis	Concentration ng/uL	A260	A280	260/280	260/230
BCM-3107-A	Infiltrating duct carcinoma, NOS	298.5	5.97	3.126	1.91	2.35
BCM-3107-B	Infiltrating duct carcinoma, NOS	318.29	6.366	3.334	1.91	2.36
BCM-3204-A	Infiltrating duct carcinoma, NOS	58.11	1.162	0.616	1.89	1.43
BCM-3204-B	Infiltrating duct carcinoma, NOS	128.36	2.567	1.338	1.92	2.21
BCM-4013-A	Infiltrating duct carcinoma, NOS	317.01	6.34	3.334	1.9	2.32
BCM-4013-B	Infiltrating duct carcinoma, NOS	674.75	13.495	7.078	1.91	2.24
BVM-4175-A	Infiltrating duct carcinoma, NOS	118.79	2.376	1.249	1.9	2.2
BVM-4175-B	Infiltrating duct carcinoma, NOS	121.3	2.426	1.281	1.89	2.18

Supplementary Table 8. The primers used for the virus detection and used as internal control.

Primers for Murine Virus Detection	Forward Primer	Reverse Primer
XMRV Gag Amplicon Size = 263	TCCGCCGAATGGCCAACTTT	GCAGATCGGGACGGAGGTTG
MLV GPP Amplicon Size = 283	GCCAGACTGGGGATCAAGCC	TGGTGGGGTGGAGTCTCAGG
XMLV Env Amplicon Size = 193	CACCCCCACCGCTCTCAAAG	GTTGTACCGAGGCTCCTGCC

Primers for Internal Control	Forward Primer	Reverse Primer
Human GAPDH Amplicon Size = 264	TCACCGGGAGGATTGGGTGT	CCCCCTAGTCCCAGGGCTTT

Reference:

1 Turner, T. H. *et al.* Characterizing the efficacy of cancer therapeutics in patient-derived xenograft models of metastatic breast cancer. **170**, 221-234 (2018).

2 Bradford, J. R. *et al.* Whole transcriptome profiling of patient-derived xenograft models as a tool to identify both tumor and stromal specific biomarkers. **7**, 20773 (2016).

3 Varley, K. E. *et al.* Recurrent read-through fusion transcripts in breast cancer. **146**, 287-297 (2014).

4 Franco, H. L. *et al.* Enhancer transcription reveals subtype-specific gene expression programs controlling breast cancer pathogenesis. **28**, 159-170 (2018).

5 Xi, Y. *et al.* Histone modification profiling in breast cancer cell lines highlights commonalities and differences among subtypes. **19**, 1-11 (2018).

6 Miller, T. E. *et al.* Transcription elongation factors represent in vivo cancer dependencies in glioblastoma. **547**, 355-359 (2017).

7 Shraibman, B., Kadosh, D. M., Barnea, E., Admon, A. J. M. & Proteomics, C. HLA peptides derived from tumor antigens induced by inhibition of DNA methylation for development of drug-facilitated immunotherapy. (2016).

8 Esteve-Codina, A. *et al.* A comparison of RNA-Seq results from paired formalin-fixed paraffin-embedded and fresh-frozen glioblastoma tissue samples. **12**, e0170632 (2017).

9 Park, N. I. *et al.* ASCL1 reorganizes chromatin to direct neuronal fate and suppress tumorigenicity of glioblastoma stem cells. **21**, 209-224. e207 (2017).

10 Li, Y. *et al.* Cytotoxic indole alkaloid 3α-acetonyltabersonine induces glioblastoma apoptosis via inhibition of DNA damage repair. **9**, 150 (2017).

11 Heiland, D. H. *et al.* Microenvironment-derived regulation of HIF signaling drives transcriptional heterogeneity in glioblastoma Multiforme. **16**, 655-668 (2018).

12 Kim, K.-T. *et al.* Single-cell mRNA sequencing identifies subclonal heterogeneity in anti-cancer drug responses of lung adenocarcinoma cells. **16**, 127 (2015).

13 Zeng, S.-X. *et al.* The phosphatidylinositol 3-kinase pathway as a potential therapeutic target in bladder Cancer. **23**, 6580-6591 (2017).

14 Wang, L. *et al.* EMT-and stroma-related gene expression and resistance to PD-1 blockade in urothelial cancer. **9**, 1-12 (2018).

15 Chen, C. *et al.* LNMAT1 promotes lymphatic metastasis of bladder cancer via CCL2 dependent macrophage recruitment. **9**, 1-18 (2018).

16 Yu, T. *et al.* Fusobacterium nucleatum promotes chemoresistance to colorectal cancer by modulating autophagy. **170**, 548-563. e516 (2017).

17 Kirby, M. K. *et al.* RNA sequencing of pancreatic adenocarcinoma tumors yields novel expression patterns associated with long-term survival and reveals a role for ANGPTL4. **10**, 1169-1182 (2016).

18 Calabretta, S. *et al.* Loss of PRMT5 promotes PDGFRα degradation during oligodendrocyte differentiation and myelination. **46**, 426-440. e425 (2018).
19 Valdes, C., Seo, P., Tsinoremas, N. & Clarke, J. J. J. o. c. b. Characteristics of cross-hybridization and cross-alignment of expression in pseudo-xenograft samples by RNA-seq and microarrays. **3**, 8 (2013).
20 Szeto, C. Y.-Y. *et al.* Integrated mRNA and microRNA transcriptome sequencing characterizes sequence variants and mRNA–microRNA regulatory network in nasopharyngeal carcinoma model systems. **4**, 128-140 (2014).
21 Mihara, T. *et al.* Linking virus genomes with host taxonomy. **8**, 66 (2016).
22 Staal, S. P. & Hartley, J. W. J. T. J. o. e. m. Thymic lymphoma induction by the AKT8 murine retrovirus. **167**, 1259-1264 (1988).
23 Diniz, J. *et al.* Characterization of two new rhabdoviruses isolated from midges (Culicoides SPP) in the Brazilian Amazon: proposed members of a new genus, Bracorhabdovirus. **151**, 2519-2527 (2006).
24 Holt, M., Shevach, E. & Punkosdy, G. J. F. i. o. Endogenous mouse mammary tumor viruses (mtv): new roles for an old virus in cancer, infection, and immunity. **3**, 287 (2013).
25 Voytek, P. & Kozak, C. J. V. HoMuLV: a novel pathogenic ecotropic virus isolated from the European mouse, Mus hortulanus. **165**, 469-475 (1988).
26 Tralka, T. S. *et al.* Murine type C retroviruses and intracisternal A-particles in human tumors serially passaged in nude mice. **71**, 591-599 (1983).
27 Luczkowiak, J., Sierra, O., González-Martín, J. J., Herrero-Beaumont, G. & Delgado, R. J. E. i. d. No xenotropic murine leukemia virus–related virus detected in fibromyalgia patients. **17**, 314 (2011).
28 Kumar, P., Nachagari, D., Fields, C., Franks, J. & Albritton, L. M. J. J. o. v. Host cell cathepsins potentiate Moloney murine leukemia virus infection. **81**, 10506-10514 (2007).
29 Hitoshi, Y. *et al.* Delayed progression of a murine retrovirus-induced acquired immunodeficiency syndrome in X-linked immunodeficient mice. **177**, 621-626 (1993).
30 Miklík, D., Šenigl, F. & Hejnar, J. J. V. Proviruses with long-term stable expression accumulate in transcriptionally active chromatin close to the gene regulatory elements: Comparison of ASLV-, HIV-and MLV-Derived Vectors. **10**, 116 (2018).
31 Bahrami, S., Duch, M. & Pedersen, F. S. J. J. o. v. Change of tropism of SL3-2 murine leukemia virus, using random mutational libraries. **78**, 9343-9351 (2004).
32 Souyri, M. *et al.* A putative truncated cytokine receptor gene transduced by the myeloproliferative leukemia virus immortalizes hematopoietic progenitors. **63**, 1137-1147 (1990).
33 Stacey, A., Arbuthnott, C., Kollek, R., Coggins, L. & Ostertag, W. J. J. o. v. Comparison of myeloproliferative sarcoma virus with Moloney murine sarcoma virus variants by nucleotide sequencing and heteroduplex analysis. **50**, 725-732 (1984).

34 Villanueva, R. A., Campbell, S. & Roth, M. J. Molecular analysis of a recombinant M-MuLV/RaLV retrovirus. *Virology* **315**, 195-208 (2003).
35 Ablashi, D. *et al.* Xenotropic Properties of an Isolate from Murine Rauscher Leukemia Virus in Primate. **5**, 223-236 (1976).
36 Zhang, Y. A. *et al.* Frequent detection of infectious xenotropic murine leukemia virus (XMLV) in human cultures established from mouse xenografts. *Cancer Biol Ther* **12**, 617-628, doi:10.4161/cbt.12.7.15955 (2011).
37 Dobin, A. *et al.* STAR: ultrafast universal RNA-seq aligner. *Bioinformatics* **29**, 15-21, doi:10.1093/bioinformatics/bts635 (2013).
38 Langmead, B. & Salzberg, S. L. Fast gapped-read alignment with Bowtie 2. *Nat Methods* **9**, 357-359, doi:10.1038/nmeth.1923 (2012).
