# Supplementary material for: Presence of complete murine viral genome sequences in patient-derived xenografts
Source: Nat Commun. 2021 Apr 1;12:2031. doi: 10.1038/s41467-021-22200-5 (PMC8017013; doi:10.1038/s41467-021-22200-5)
Supplement: Supplementary file 3 — Description of Additional Supplementary Files [file 41467_2021_22200_MOESM3_ESM.pdf]

Description of Additional Supplementary Information

File Name: Supplementary Data 1

Description: The list of detected viruses in all samples.

File Name: Supplementary Data 2

Description: The assembly of chimeric reads containing both virus and human sequences.
