# Supplementary material for: Presence of complete murine viral genome sequences in patient-derived xenografts
Source: Nat Commun. 2021 Apr 1;12:2031. doi: 10.1038/s41467-021-22200-5 (PMC8017013; doi:10.1038/s41467-021-22200-5)
Supplement: Supplementary file 6 — Reporting Summary [file 41467_2021_22200_MOESM6_ESM.pdf]

nature research

Corresponding author(s): W. Jim Zheng
Zhiqiang An

Last updated by author(s): 1/27/2021

Reporting Summary

Nature Research wishes to improve the reproducibility of the work that we publish. This form provides structure for consistency and transparency in reporting. For further information on Nature Research policies, see Authors & Referees and the Editorial Policy Checklist .

Statistics

For all statistical analyses, confirm that the following items are present in the figure legend, table legend, main text, or Methods section.

n/a	Confirmed	
☐	☒	The exact sample size (*n*) for each experimental group/condition, given as a discrete number and unit of measurement
☐	☒	A statement on whether measurements were taken from distinct samples or whether the same sample was measured repeatedly
☐	☒	The statistical test(s) used AND whether they are one- or two-sided<br>*Only common tests should be described solely by name; describe more complex techniques in the Methods section.*
☒	☐	A description of all covariates tested
☐	☒	A description of any assumptions or corrections, such as tests of normality and adjustment for multiple comparisons
☐	☒	A full description of the statistical parameters including central tendency (e.g. means) or other basic estimates (e.g. regression coefficient) AND variation (e.g. standard deviation) or associated estimates of uncertainty (e.g. confidence intervals)
☐	☒	For null hypothesis testing, the test statistic (e.g. *F*, *t*, *r*) with confidence intervals, effect sizes, degrees of freedom and *P* value noted<br>*Give P values as exact values whenever suitable.*
☒	☐	For Bayesian analysis, information on the choice of priors and Markov chain Monte Carlo settings
☒	☐	For hierarchical and complex designs, identification of the appropriate level for tests and full reporting of outcomes
☐	☒	Estimates of effect sizes (e.g. Cohen's *d*, Pearson's *r*), indicating how they were calculated

Our web collection on statistics for biologists contains articles on many of the points above.

Software and code

Policy information about availability of computer code

Data collection	SRA: (https://trace.ncbi.nlm.nih.gov/Traces/sra/)<br>Virus-Host DB (https://www.genome.jp/virushostdb/)
Data analysis	BoxPlotR: (http://shiny.chemgrid.org/boxplotr/, Latest commit 59180dc on Jul 23, 2015)<br>Bowtie 2: (http://bowtie-b io. sou rceforge. net/bowtie2/ind ex.sh tml)<br>Circoletto (http://tools.bat.infspire.org/circoletto/)<br>EdgeR: version v3.14.0 (https://bioconductor.org/packages/release/bioc/html/edgeR.html)<br>GeneCards (https://www.genecards.org/)<br>HISAT2: version 2.1.0 (http://daehwankimlab.github.io/hisat2/)<br>HTSeq: version 0.7.2. (https://pypi.org/project/HTSeq/)<br>Megahit (https://github.com/voutcn/megahit)<br>MUSCLE: inmplemeted in MEGA7 (https://www.megasoftware.net)<br>NCBI taxonomy databases (https://www.ncbi.nlm.nih.gov/guide/taxonomy/)<br>PCA performed in Omicshare (http://www.omicshare.com/tools/Home/Soft/pca):<br>PhyML: version PhyML 3.0 (http://www.atgc-montpellier.fr/phyml/)<br>R language package: version R 3.6.0 (https://cran.r-project.org/bin/windows/base/old/3.6.0/)<br>READSCAN: version readscan-0.5 (https://github.com/raeece/readscan)<br>Samtools: version samtools 1.6 (https://github.com/samtools/)<br>SMS (Smart Model Selection in PhyML) (http://www.atgc-montpellier.fr/sms/)<br>SRA Toolkit: version sratoolkit/2.8.2 (https://github.com/ncbi/sra-tools)<br>sp_enrichmentPlot.sh: (https://github.com/Tong-Chen/s-plot/blob/master/sp_enrichmentPlot.sh)<br>STAR: (https://githu b.com/alexdobin/STAR, version 2.7)<br>Trimmomatic: version Trimmomatic-0.39 (https://github.com/timflutre/trimmomatic)

UCSC Genome Browser (https://genome.ucsc.edu)
Virus-Clip: (http://web.hku.hk/~dwhho/Virus-Clip.zi p)
WebGestalt (http://www.webgestalt.org/)

For manuscripts utilizing custom algorithms or software that are central to the research but not yet described in published literature, software must be made available to editors/reviewers. We strongly encourage code deposition in a community repository (e.g. GitHub). See the Nature Research guidelines for submitting code & software for further information.

Data

Policy information about availability of data

All manuscripts must include a data availability statement. This statement should provide the following information, where applicable:
- Accession codes, unique identifiers, or web links for publicly available datasets
- A list of figures that have associated raw data
- A description of any restrictions on data availability

All the data used in this work were obtained from Sequence Reads Archive (SRA: https://trace.ncbi.nlm.nih.gov/Traces/sra/.). These data sets are listed in Supplementary Table 2 with their unique accession numbers, and can be freely downloaded using the accession numbers. The remaining data are available within the Article, Supplementary Information or available from the authors upon request.

All the virus information was obtained from Virus-Host DB (https://www.genome.jp/virushostdb/).

Field-specific reporting

Please select the one below that is the best fit for your research. If you are not sure, read the appropriate sections before making your selection.

☒ Life sciences ☐ Behavioural & social sciences ☐ Ecological, evolutionary & environmental sciences

For a reference copy of the document with all sections, see nature.com/documents/nr-reporting-summary-flat.pdf

Life sciences study design

All studies must disclose on these points even when the disclosure is negative.

Sample size

Since this is a data re-analysis project, the sample size was determined by each original experiment. Therefore, no sample size calculation was involved.

Four independent PDX samples were used to validate the presence of murine leukemia viruses, exceeding the conventional experimental validation by triplicates. No sample size calculation was involved.

Data exclusions

No data were excluded in the analysis

Replication

Since this is a data re-analysis project, we adhere to the original experimental design of the source experiments. The finding of murine leukemia viruses are confirmed by the presence of viruses in 177 of the 184 data sets.

All the PCR validation experiments were performed twice with 4 different samples.

Randomization

Not applicable

Blinding

Not applicable

Reporting for specific materials, systems and methods

We require information from authors about some types of materials, experimental systems and methods used in many studies. Here, indicate whether each material, system or method listed is relevant to your study. If you are not sure if a list item applies to your research, read the appropriate section before selecting a response.

Materials & experimental systems

n/a	Involved in the study
☒	☐ Antibodies
☒	☐ Eukaryotic cell lines
☒	☐ Palaeontology
☒	☐ Animals and other organisms
☒	☐ Human research participants
☒	☐ Clinical data

Methods

n/a	Involved in the study
☒	☐ ChIP-seq
☒	☐ Flow cytometry
☒	☐ MRI-based neuroimaging
